# The c-Jun and JunB transcription factors facilitate the transit of classical Hodgkin lymphoma tumour cells through G_**1**_

**DOI:** 10.1038/s41598-018-34199-9

**Published:** 2018-10-30

**Authors:** Jingxi Zhang, Zuoqiao Wu, Anton Savin, Mihye Yang, Ying-Han R. Hsu, Eugeniu Jantuan, Julinor T. C. Bacani, Robert J. Ingham

**Affiliations:** 1 Department of Medical Microbiology and Immunology and Li Ka Shing Institute of Virology, University of Alberta, Katz Group Centre for Pharmacy and Health Research, University of Alberta, Edmonton, AB T6G 2E1 Canada; 2 Department of Laboratory Medicine and Pathology, University of Alberta, Edmonton, AB T6G 2B7 Canada

## Abstract

Classical Hodgkin Lymphoma (cHL) is primarily a B cell lymphoid neoplasm and a member of the CD30–positive lymphomas. cHL and the other CD30–positive lymphomas are characterized by the elevated expression and/or constitutive activation of the activator protein-1 (AP-1) family transcription factors, c-Jun and JunB; however, the specific roles they play in the pathobiology of cHL are unclear. In this report we show that reducing either c-Jun or JunB expression with short-hairpin RNAs (shRNAs) reduced the growth of cHL cell lines *in vitro* and *in vivo*, primarily through impairing cell cycle transition through G_1_. We further investigated the effect of c-Jun and JunB knock-down on proliferation in another CD30–positive lymphoma, anaplastic lymphoma kinase-positive, anaplastic large cell lymphoma (ALK+ ALCL). We found that JunB knock-down in most ALK+ ALCL cell lines examined also resulted in reduced proliferation that was associated with a G_0_/G_1_ cell cycle defect. In contrast, c-Jun knock-down in multiple ALK+ ALCL cell lines had no effect on proliferation. In summary, this study directly establishes that both c-Jun and JunB play roles in promoting HRS cell proliferation. Furthermore, we demonstrate there are similarities and differences in c-Jun and JunB function between cHL and ALK+ ALCL.

## Introduction

Classical Hodgkin lymphoma (cHL) is primarily a B cell lineage lymphoma that accounts for 95% of all Hodgkin lymphoma^1^. It is characterized histopathologically by hallmark mononuclear Hodgkin and multinuclear Reed-Sternberg (HRS) tumour cells that are derived from germinal center B cells^2^. HRS cells express the tumour necrosis factor receptor family member, CD30 (*TNFRSF8*), which is common to cHL and other CD30–positive lymphomas such as anaplastic lymphoma kinase-positive, anaplastic large cell lymphoma (ALK+ ALCL), anaplastic lymphoma kinase-negative, anaplastic large cell lymphoma (ALK− ALCL), cutaneous ALCL, and CD30–positive diffuse large B cell lymphoma^3,4^. An intriguing feature of cHL is that the tumour cells make up only a small proportion of the tumour mass with apparently normal leukocytes constituting the vast majority of cells^1,2^. The interactions between tumour infiltrating leukocytes and HRS cells are multi-faceted. HRS cells directly or indirectly attract leukocytes to benefit from cytokines produced by these cells, while simultaneously creating an immunosuppressive microenvironment that prevents tumour cell killing^2,5^.

The dysregulation of multiple transcription factors is a hallmark of HRS cells. For example, NF-κB^6,7^, STATs^8,9^, GATA3^10,11^, and IRF5^12^ play important roles in cHL pathogenesis, whereas PU.1 (*SPI1*)^13,14^, EBF1^15,16^, and FOXO1^17^ function as tumour suppressors in this lymphoma and are repressed. The elevated expression and/or activation of the activator protein-1 (AP-1) family transcription factors, c-Jun (*JUN*) and JunB, are also found in cHL^18–21^ as well as other CD30–positive lymphomas^19,20,22–25^. AP-1 proteins consist of members of the Jun, Fos, ATF, and Maf subfamilies^26–28^, and they form heterodimers between the family members and some can also homodimerize^28^. Collectively, these transcription factors regulate the expression of numerous genes including those associated with proliferation^26,27^, apoptosis^26,27^, differentiation^29^, and the immune response^29^.

Several transcriptional targets of c-Jun and/or JunB have been described in cHL. These include *CD30*^19,30^, *c-Myc* (*MYC*)^31^, as well as genes associated with immune evasion such as *Programmed Cell Death 1 Ligand 1* (*PD-L1; CD274*)^32^ and *Galectin-1 (LGALS1)*^33^. c-Jun has also been suggested to promote the transcription of *Lymphotoxin-α (LTA)* in HRS cells, which is important for shaping the cHL tumour microenvironment^34^. Moreover, since AP-1 binding sites are enriched in accessible chromatin in HRS cells^12^, there are likely many undescribed c-Jun/JunB–regulated genes that are important in the pathobiology of this lymphoma.

An important question that has not been fully addressed is whether these related transcription factors have largely overlapping or distinct functions in cHL. For example, *CD30* has been described as a JunB–specific target^19,30^, whereas both c-Jun and JunB have been suggested to promote *PD-L1* transcription^32^. Likewise, while AP-1 activity appears to be required for cHL proliferation, it is unclear whether this phenotype can be directly attributed to c-Jun and/or JunB. Leventaki *et al*. showed that pharmacological inhibition of the c-Jun activator, Jnk, resulted in a reduced growth rate in cHL cell lines that was characterized by an increased percentage of cells in G_2_/M^21^. However, Jnk has many substrates, including JunB^35^, and the Jnk inhibitor used in this study, SP600125, targets other protein kinases^36^. Thus, it is unclear what SP600125 is inhibiting in these cells to cause this growth defect. In addition, Mathas and colleagues demonstrated that over-expression of A-Fos, a dominant negative c-Fos construct, reduced the growth rate of the L-428 cHL cell line^18^; but since c-Fos can dimerize with multiple AP-1 proteins^28^, this phenotype cannot be directly attributed to inhibition of c-Jun and/or JunB.

In this study, we investigated whether c-Jun and JunB have specific roles in regulating cHL proliferation using short-hairpin RNAs (shRNAs) to individually knock-down the expression of these transcription factors. Furthermore, we used this same approach to investigate whether stable c-Jun or JunB knock-down results in a similar phenotype in another CD30-positive lymphoma, ALK+ ALCL.

## Results

### shRNA–mediated knock-down of either c-Jun or JunB reduced the growth rate of cHL cell lines *in vitro* and *in vivo*

**Figure 1:**
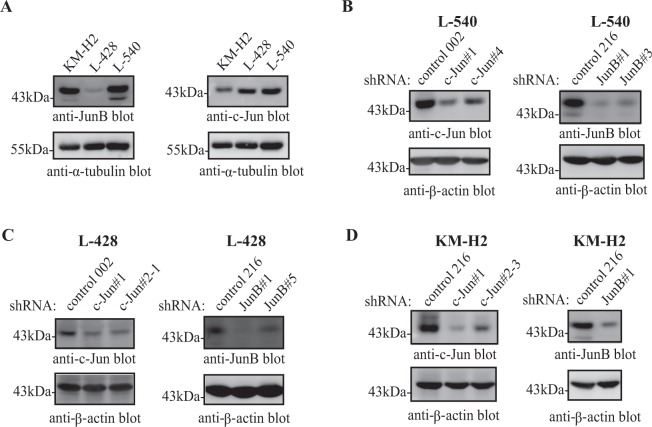
c-Jun/JunB knock-down in cHL cell lines. (**A**) Western blots examining the expression levels of c-Jun and JunB in the indicated cHL cell lines. Note: the two blots are the same lysates run on one gel that was cut prior to probing. (**B**–**D**) Western blots demonstrating the efficacy of c-Jun/JunB knock-down in L-540 (**B**), L-428 (**C**) and KM-H2 (**D**) cells stably expressing the indicated shRNAs. Molecular mass markers (in kDa) are indicated to the left of western blots.

To investigate the specific function of c-Jun and JunB in cHL, we stably knocked-down their expression in the L-540, L-428, and KM-H2 cHL cell lines. These cell lines were chosen because they express different levels of c-Jun and JunB (Fig. 1A), and collectively represent two distinct cHL histological subtypes^37^. We initially screened multiple c-Jun/JunB shRNAs to identify those that most efficiently reduced c-Jun/JunB expression in each cell line (results not shown). For each cell line, we were able to reduce the expression of each of these transcription factors with two distinct shRNAs (Fig. 1B–D) with the exception of KM-H2 cells where we were only able to identify one shRNA that significantly reduced JunB expression (results not shown). Moreover, c-Jun/JunB expression was also reduced in knock-down cells relative to a distinct control shRNA (Supplementary Fig. S1).

**Figure 2:**
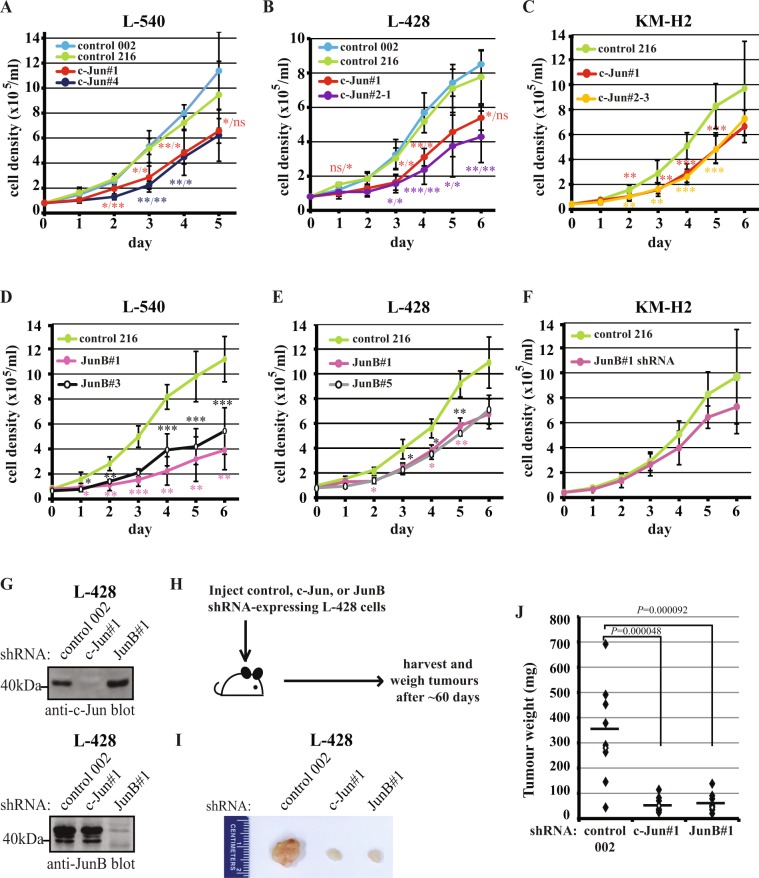
c-Jun/JunB knock-down in cHL cell lines results in a reduced growth rate. **(A**–**F**) Growth curves examining the effect of c-Jun (**A**–**C**) or JunB (**D**–**F**) knock-down in cHL cell lines expressing the indicated shRNAs. The results represent the average and standard deviation of four (**A**,**B**), five (**C**,**D**,**F**) or three (**E**) independent experiments from two separate infections. Note: the control shRNA data in (**C**,**F**) is the same because c-Jun and JunB knock-down cells were examined together in the same experiments. (**G**) Western blots showing c-Jun and JunB levels in L-428 cells expressing the indicated shRNAs used in the mouse xenograft experiments. Note: the two blots are the same lysates run on one gel that was cut prior to probing. Molecular mass markers (in kDa) are indicated to the left of western blots. (**H**) Ten mice were injected with each of the shRNA–expressing cell lines shown in (**G**). Fifty-nine days after injection, mice were euthanized and tumours were excised. Pictures showing representative median sized tumours from each group (**I**), and plot showing the weight (in mg) of tumours isolated from mice (**J**). The average tumour weight in each group is represented by the horizontal line and the open dot indicates the corresponding tumour in (**I**). The results of the mice xenograft experiments are representative of two independent experiments. Note: in the experiment shown one mouse in the control shRNA group was euthanized prior to day 59 because the tumour had reached an unacceptable size, and we could not find the tumour in one mouse in the JunB shRNA group. *P* values were obtained by performing ANOVA with Tukey’s *post hoc* test comparing controls to c-Jun/JunB shRNA–expressing cells with the exception that a two-tailed *t* test was performed in (**F**). comparing control to JunB shRNA–expressing cells. ns; not significant, *P < 0.05, **P < 0.01, ***P < 0.001. In (**A**,**B**), the first *P* value is the knock-down compared to control 002 and the second is compared to control 216.

Knock-down of either c-Jun or JunB resulted in a decreased growth rate in all cell lines (Fig. 2A–E; Supplementary Fig. S1); albeit, while the growth rate of JunB shRNA–expressing KM-H2 cells was consistently reduced in each experiment, the averaged results did not reach statistical significance (Fig. 2F). We also examined whether c-Jun and JunB were required for the growth of cHL cell lines *in vivo*. Control, c-Jun, or JunB shRNA–expressing L-428 cells (Fig. 2G) were injected into immunocompromised mice and tumour growth was examined (Fig. 2H). Tumours formed from cells with decreased c-Jun or JunB expression were significantly reduced in size (Fig. 2I) and weight (Fig. 2J) compared to tumours formed from control shRNA–expressing cells. Taken together, these results demonstrate that both c-Jun and JunB promote the growth of cHL cell lines *in vitro* and *in vivo*, but the impact of knock-down of these transcription factors varies amongst the cell lines.

### Stable knock-down of c-Jun or JunB in cHL cell lines resulted in a prolonged G_0_/G_1_

**Figure 3:**
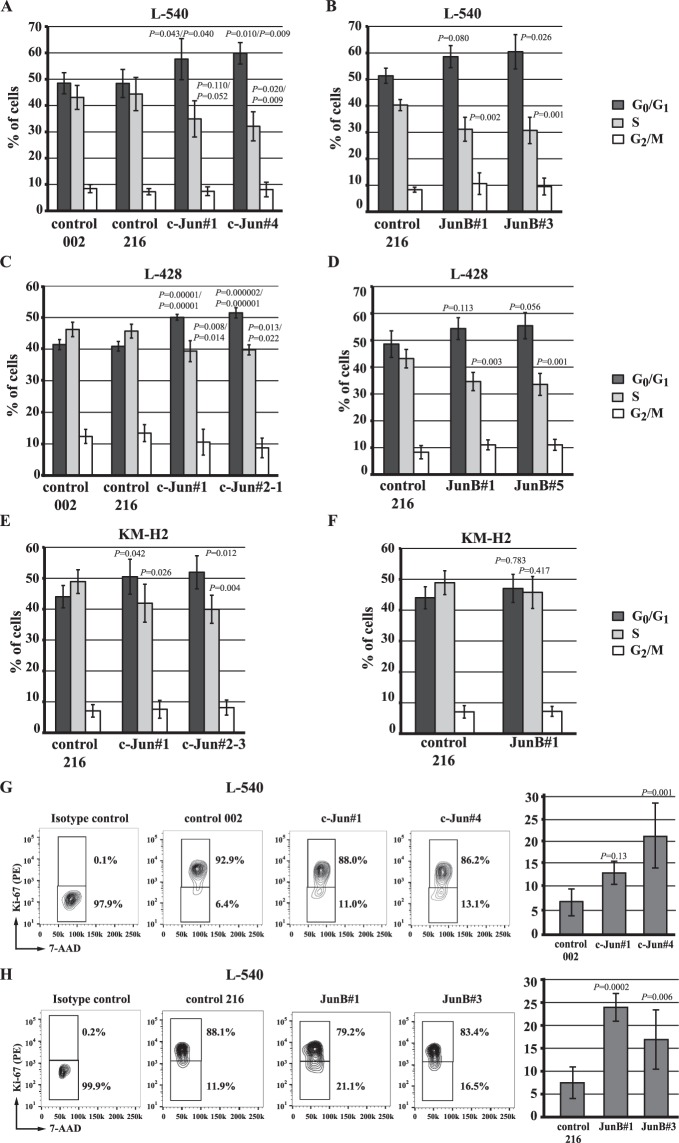
c-Jun/JunB knock-down results in a similar cell cycle alteration within cHL cell lines. The percentage of cells at each stage of the cell cycle was measured by BrdU/7-AAD double staining of L-540 (**A**,**B**), L-428 (**C**,**D**) or KM-H2 (**E**,**F**) cells expressing control, c-Jun, or JunB shRNAs. The results represent the average and standard deviation of at least four independent experiments from two separate infections. (**G**,**H**) Representative flow cytometry plots and summary of Ki-67 expression within the G_0_/G_1_ population of L-540 cells expressing the indicated shRNAs. The summaries represent the average and standard deviation of five independent experiments from at least two separate infections. Note: the control shRNA data in (**E**,**F**) is the same because c-Jun and JunB knock-down cells were examined together in the same experiments. *P* values were obtained by performing ANOVA with Tukey’s *post hoc* test comparing the c-Jun/JunB knock-down cells with control shRNA–expressing cells. A two-tailed *t* test was performed in (**F**). comparing control and JunB shRNA–expressing cells. In (**A**,**B**), the first *P* value is the knock-down compared to control 002 and the second is compared to control 216.

We next examined whether the decreased growth rate in the knock-down cell lines was due to a proliferation defect. BrdU and 7-AAD double staining experiments revealed that knocking down c-Jun or JunB expression in all cell lines resulted in a decreased percentage of cells in S phase and a concomitant increase in the percentage in G_0_/G_1_ (Fig. 3A–F; Supplementary Figs S1 and S2), although this did not always reach statistical significance and the changes in JunB knock-down KM-H2 cells were modest (Fig. 3F). Notably, with the exception of some early time points in some JunB knock-down L-540 cells, in particular JunB#1 shRNA, apoptosis was not a factor contributing to the reduced growth rate of cells (Supplementary Fig. S3).

We used the percentages of cells in each stage of the cell cycle (Fig. 3) and doubling times estimated from the growth curves in Fig. 2 to gain an appreciation of the time cells spent in each stage of the cell cycle^38^ (Table 1). We excluded the JunB shRNA–expressing L-540 cells because of the apoptosis observed in these cells early in the growth curve experiments. The most consistent difference observed was a statistically significant prolonged G_0_/G_1_ which was increased ~20–80% in the c-Jun/JunB knock-down cell lines (Table 1). Notably, the results were consistent when knock-down cells were compared to either control shRNA-expressing cells.

**Table 1:** c-Jun or JunB knock-down in cHL cell lines is associated with a prolonged G_0_/G_1_.

L-540	Control 002	Control 216	c-Jun#1	c-Jun#4
Doubling time (h)	29	34	40	43
G_0_/G_1_ (h)	14.1 +/− 1.2	16.5 +/− 1.8	23.1 +/− 3.1***/***	25.7 +/− 1.8***/***
S (h)	12.5 +/− 1.3	15.1 +/− 2.2	14.0 +/− 2.8*	13.8 +/− 2.4
G_2_/M (h)	2.4 +/− 0.4	2.5 +/− 0.4	3.0 +/− 0.7	3.5 +/− 1.2
**L-428**	**Control 002**	**Control 216**	**c-Jun#1**	**c-Jun#2-1**
Doubling time (h)	36	37	42	49
G_0_/G_1_ (h)	14.9 +/− 0.6	15.1 +/− 0.6	21.3 +/− 0.4***/***	25.2 +/− 0.8***/***
S (h)	16.6 +/− 0.8	16.9 +/− 0.8	16.5 +/− 1.4	19.5 +/− 0.8**/*
G_2_/M (h)	4.5 +/− 0.8	5.0 +/− 1.0	4.4 +/− 1.7	4.3 +/− 1.5
**L-428**	**Control 216**	**JunB#1**	**JunB#5**	
Doubling time (h)	34	37	40	
G_0_/G_1_ (h)	16.5 +/− 1.7	19.3 +/− 1.4**	21.3 +/− 1.9***	
S (h)	14.7 +/− 1.2	13.3 +/− 1.7	13.8 +/− 1.9	
G_2_/M (h)	2.8 +/− 0.8	4.5 +/− 0.4*	4.8 +/− 0.6**	
**KM-H2**	**Control 216**	**c-Jun#1**	**c-Jun#2-3**	**JunB#1**
Doubling time (h)	28	35	34	31
G_0_/G_1_ (h)	12.3 +/− 1.0	17.7 +/− 2.0***	17.7 +/− 1.8***	14.6 +/− 1.4*
S (h)	13.7 +/− 1.1	14.7 +/− 2.2	13.6 +/− 1.6	14.2 +/− 1.6
G_2_/M (h)	2.0 + /−0.6	2.7 +/− 1.0	2.8 +/− 0.8	2.3 +/− 0.5

Approximate doubling time and time spent in each stage of the cell cycle was determined for L-540, L-428 and KM-H2 cells expressing control, c-Jun or JunB shRNA as described in the Methods. The results represent the average and standard deviation of at least four independent experiments. *P* values were obtained by performing ANOVA with Tukey’s *post hoc* test comparing the c-Jun/JunB knock-down cells with control shRNA–expressing cells. When two *P* values are given, the first is compared to control 002 and the second is to control 216. *P < 0.05, **P < 0.01, ***P < 0.001.

We further investigated whether the prolonged G_0_/G_1_ might be due to some cells leaving the cell cycle. Ki-67 (*MKI67*) is expressed in cycling cells, but absent in G_0_ cells^39^ or reduced in those that have recently left cycle^40^. Therefore, we examined Ki-67 staining within the G_0_/G_1_ population and observed a greater proportion of cells with low Ki-67 staining when c-Jun (Fig. 3G) or JunB (Fig. 3H) was knocked-down in L-540 cells; albeit the results in c-Jun knock-down cells were less impressive and the difference in c-Jun#1 knock-down cells did not reach statistical significance. Taken together, these findings indicate that knock-down of either c-Jun or JunB in cHL cell lines is associated with a prolonged G_0_/G_1_, and that some knock-down cells, particularly JunB knock-down cells, may even leave the cell cycle.

### Stable knock-down of JunB, but not c-Jun, reduced ALK+ ALCL proliferation

Elevated expression and/or the constitutive activation of c-Jun and JunB are common to other CD30–positive lymphomas including ALK+ ALCL^19,20,22,23^, ALK− ALCL^20,24^, cutaneous ALCL^20,24,25^, and CD30–positive diffuse large B cell lymphoma^20,24^. Transient short interfering RNA (siRNA)–mediated knock-down of JunB in ALK+ ALCL cell lines was found to decrease proliferation^22,41^, whereas c-Jun knock-down has been suggested to either impair^23^ or not affect proliferation^22^. We wanted to examine whether stable shRNA–mediated knock-down of c-Jun or JunB in ALK+ ALCL cell lines had a similar effect on proliferation as observed in cHL cell lines.

**Figure 4:**
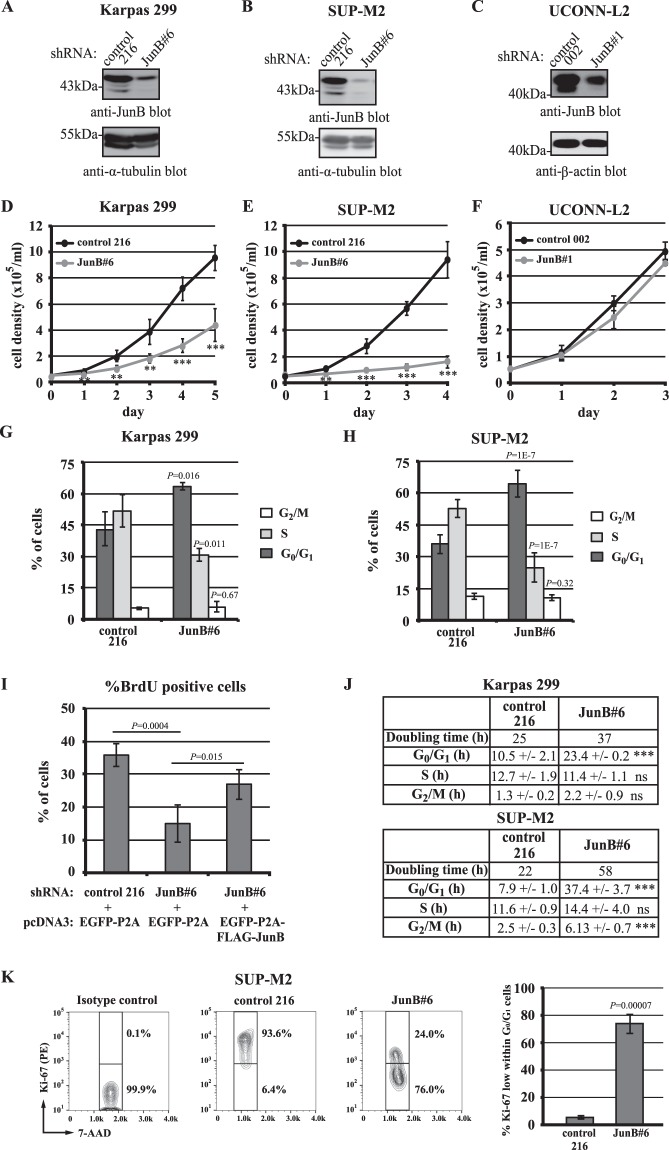
shRNA–mediated knock-down of JunB in ALK+ ALCL cell lines results in reduced proliferation. Western blots showing JunB levels (**A**–**C**) and growth curves (**D**–**F**) of the indicated cell lines expressing either control or JunB shRNA. Growth curves represent five experiments from three infections (**D**), five experiments from five infections (**E**), and three experiments from one infection (**F**). Note: JunB#6 and JunB#1 shRNAs overlap in their seed sequence and are not distinct. BrdU/7-AAD double staining of Karpas 299 (**G**) or SUP-M2 (**H**) cells expressing control or JunB shRNA. Results represent the average and standard deviation of three independent experiments from three infections for Karpas 299 cells, and eight independent experiments from six infections for SUP-M2 cells. (**I**) Bar graph showing the percentage of cells in S phase (% BrdU positive cells) observed when control shRNA or JunB shRNA–expressing Karpas 299 cells were transfected with plasmids expressing either EGFP-P2A or EGFP-P2A-FLAG-JunB. The results represent the average and standard deviation of four independent experiments. (**J**) Approximate doubling times and time spent in each stage of the cell cycle was determined for Karpas 299 and SUP-M2 cells expressing control or JunB shRNA. (**K**) Representative flow cytometry data and summary of Ki-67 expression within the G_0_/G_1_ population of SUP-M2 cells expressing control or JunB shRNA. The summary represents the average and standard deviation of three independent experiments from two separate infections. *P* values were obtained by performing independent, two-tailed *t* tests comparing the JunB knock-down to control shRNA–expressing cells (**D**–**F** and **K**) or between JunB knock-down cells with or without JunB cDNA (**I**). ANOVA with Tukey’s *post hoc* test was performed in (**I**). *P < 0.05, **P < 0.01, ***P < 0.001. Molecular mass markers (in kDa) are indicated to the left of western blots.

We found that JunB knock-down (Fig. 4A–C; Supplementary Fig. S4) was associated with a reduced growth rate in the Karpas 299 and SUP-M2 ALK+ ALCL cell lines, but had no effect on the growth rate of the UCONN-L2 cell line (Fig. 4D–F; Supplementary Fig. S4). Similar to what was observed in cHL cell lines, JunB knock-down in Karpas 299 and SUP-M2 cells was associated with a decreased percentage of cells in S phase and a marked increase in the percentage in G_0_/G_1_ (Fig. 4G,H; Supplementary Fig. S4). No significant apoptosis was observed in Karpas 299 or SUP-M2 cells when JunB was knocked-down (Fig. S3E,F). We did not observe a proliferation defect with additional JunB shRNAs, but these were not able to reduce JunB expression to the same extent as JunB#6 and JunB#1 shRNAs (results not shown) which overlap with respect to their seed sequences. We postulated that the lack of observed phenotype with these additional JunB shRNAs was likely due to poor silencing. Therefore, to rule out that decreased proliferation observed in cells expressing JunB#6 shRNA was due to off-targeting, we examined whether we could rescue the proliferation defect in JunB knock-down cells through the addition of a FLAG epitope–tagged JunB cDNA (Supplementary Fig. S5). Of note, this shRNA targets the 3′ untranslated region of the JunB mRNA, and therefore would not be expected to target the introduced JunB cDNA. Transfection of the FLAG–tagged JunB cDNA into JunB shRNA–expressing cells resulted in an increased percentage of cells in S phase (Fig. 4I), arguing that the proliferation defect in these cells is due to decreased JunB expression and not the shRNA targeting some other gene(s). Calculating the doubling times from the growth curves and relating this to the cell cycle distribution data revealed that JunB knock-down in both Karpas 299 and SUP-M2 cells resulted in a significant increase in the time spent in G_0_/G_1_ (Fig. 4J). In addition, JunB knock-down in SUP-M2 cells was associated with a prominent population of Ki67–low cells (Fig. 4K). Thus, our results demonstrate that JunB knock-down in the majority of ALK+ ALCL cell lines tested was associated with a prolonged G_0_/G_1_ similar to what was observed in c-Jun/JunB knock-down cHL cell lines.

**Figure 5:**
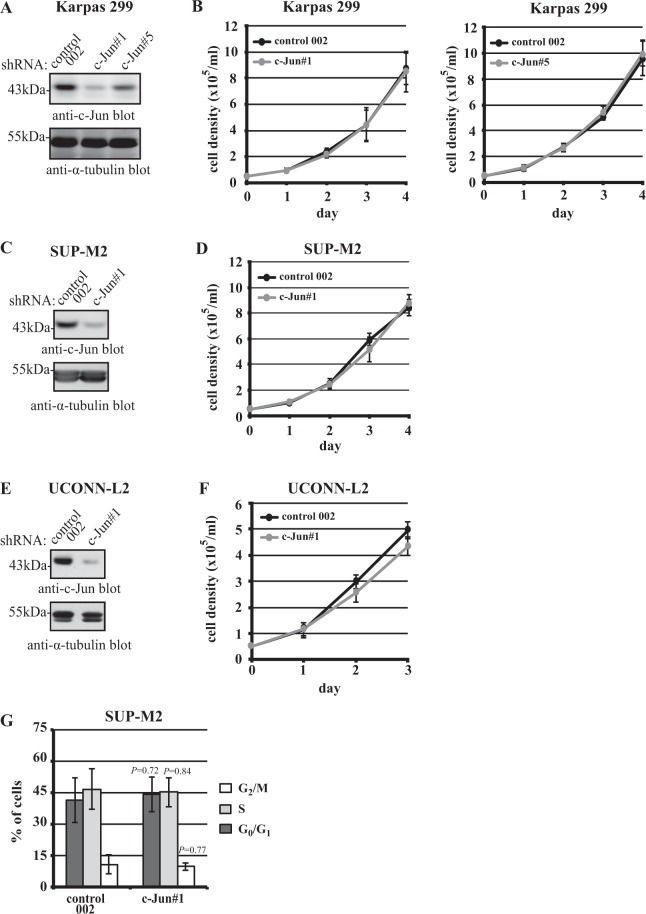
shRNA–mediated knock-down of c-Jun in ALK+ ALCL cell lines does not impair proliferation. Western blots showing c-Jun levels (**A**,**C**, and **E**) and growth curves (**B**,**D**, and **F**) of the indicated cell lines expressing either control or c-Jun shRNA. Growth curves represent four experiments from four infections (c-Jun#1) and five experiments from three experiments (c-Jun#5) (**B**), three experiments from three infections (**D**), and three experiments from one infection (**F**). Although, not included in the growth curve shown, c-Jun knock-down in UCONN-L2 had no effect on proliferation in other infections. (**G**) The percentage of cells in each stage of the cell cycle was measured by BrdU/7-AAD double staining for SUP-M2 cells expressing either control or c-Jun shRNA. The results represent three experiments from three infections. No statistically significant differences in growth rate or cell cycle distribution were observed between any c-Jun knock-down cells and respective control shRNA–expressing cells. Molecular mass markers (in kDa) are indicated to the left of western blots.

In contrast to JunB, shRNA–mediated knock-down of c-Jun in multiple ALK+ ALCL cell lines had no observable effect on growth rate (Fig. 5A–F), and no alteration of the cell cycle was observed in c-Jun shRNA–expressing SUP-M2 cells (Fig. 5G). These results demonstrate an important distinction in the consequence of reducing c-Jun expression in cHL and ALK+ ALCL.

### Increased expression of G1 CDK inhibitors correlated with the G0/G1 cell cycle defect

**Figure 6:**
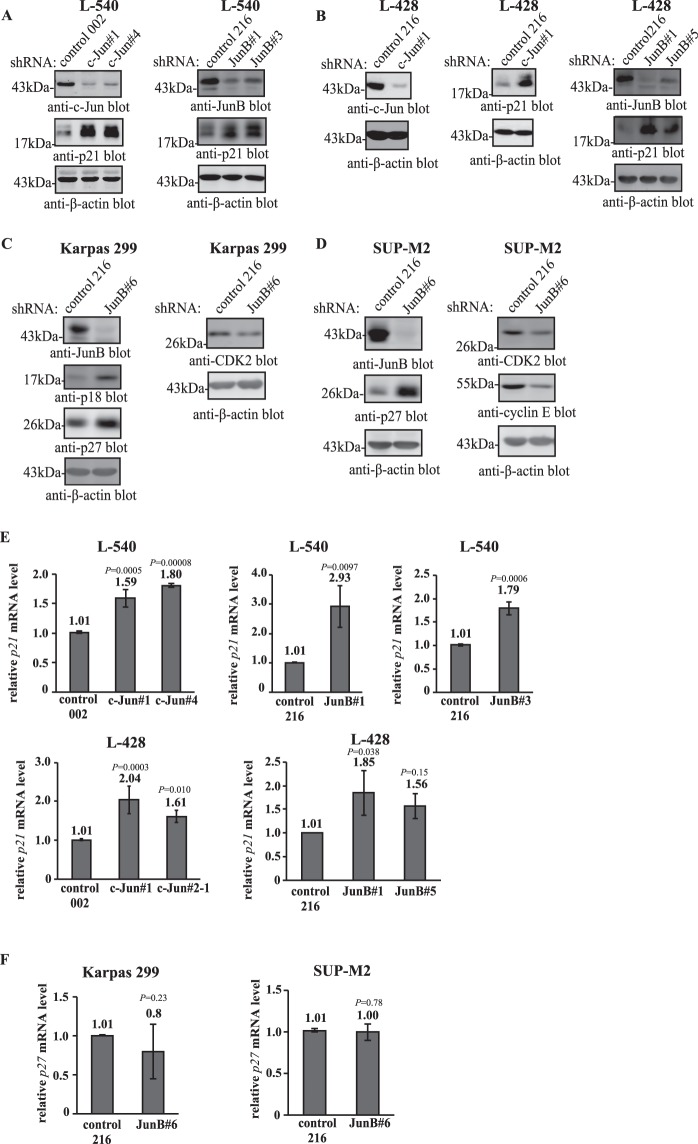
Dysregulation of G_1_ regulators in c-Jun/JunB knock-down cell lines. Western blots demonstrating the differential expression of p21^*Cip1/Waf1*^ protein levels in L-540 (**A**) and L-428 (**B**) cHL cell lines expressing the indicated shRNAs. In (**B**), the two membranes with control and c-Jun#1 samples are the same lysates run on two different gels. (**C**,**D**) Western blots comparing the expression of the indicated cell cycle regulators between control and JunB shRNA–expressing Karpas 299 (**C**) and SUP-M2 (**D**) cells. In both (**C**,**D**), each vertical column of blots represents the same lysates run out on different gels. Molecular mass markers (in kDa) are indicated to the left of western blots. qRT-PCR data examining *p21*^*Cip1/Waf1*^ (**E**) or *p27*^*Kip1*^ (**F**) mRNA expression in the indicated cell lines. The results represent the average and standard deviation of at least 3 independent experiments from at least two infections. *P* values were obtained by performing ANOVA with Tukey’s *post hoc* test when c-Jun/JunB knock-down cells with two shRNAs with control shRNA–expressing cells or independent, two-tailed *t* tests when comparing JunB knock-downs with one shRNA to control shRNA–expressing cells.

Our results show that reducing c-Jun or JunB expression in cHL cell lines, or JunB expression in most ALK+ ALCL cell lines, is associated with a defect in the ability of cells to transit through G_1_. We next wanted to investigate whether this correlated with the dysregulation of known G_1_ regulators, and if so, whether the alterations were similar amongst the different cell lines and lymphomas. To that end, the expression of several G_1_ cyclins (Cyclins D2, D3, and E), cyclin-dependent kinases (CDKs; CDKs 2, 4 and 6), and CDK inhibitors (p21^*Cip1/Waf1*^, p27^*Kip1*^, and p18^*Ink4C*^) were compared between control and c-Jun/JunB shRNA–expressing cells. We found that the levels of some of the proteins were similar between control and c-Jun/JunB knock-down cells, and for others it was inconclusive whether they were altered when c-Jun/JunB was knocked-down (results not shown). Nonetheless, in the cHL cell lines, we observed an up-regulation of the p21^*Cip1/Waf1*^ (*CDKNIA*) CDK inhibitor (Fig. 6A,B) when c-Jun or JunB was knocked-down; although this was not as reproducibly convincing in c-Jun#2-1 shRNA–expressing cells (results not shown). In the ALK+ ALCL cell lines, we also observed an up-regulation of CDK inhibitors when JunB was knocked-down including increased expression of p27^*Kip1*^(*CDKNIB*) in JunB shRNA–expressing Karpas 299 and SUP-M2 cells, and p18^*Ink4C*^ (*CDKN2C*) in Karpas 299 cells **(**Fig. 6C,D). We also detected alterations in other G_1_ regulators in the ALK+ ALCL cell lines when JunB was knocked-down including decreased expression of cyclin-dependent kinase 2 (CDK2) in both Karpas 299 and SUP-M2 cells, as well as reduced levels of cyclin E in SUP-M2 cells.

Up-regulation of p21^*Cip1/Waf1*^ in c-Jun/JunB knock-down cHL cell lines and up-regulation of p27^*Kip1*^ in the JunB knock-down ALK+ ALCL cell lines were the most prominently altered G_1_ regulators at the protein level. We next investigated whether increased transcription might account for some of the increases in p21^*Cip1/Waf1*^ and p27^*Kip1*^ protein levels. qRT-PCR experiments demonstrated an increase in *p21*^*Cip1/Waf1*^ gene transcription in c-Jun and JunB knock-down L-428 and L-540 cells, although this did not reach statistical significance for L-428 cells expressing JunB shRNA #5 **(**Fig. 6E). In contrast, *p27*^*Kip1*^ gene expression was not significantly different between control and JunB shRNA-expressing SUP-M2 cells and was not increased, albeit quite variable, in Karpas 299 cells when JunB was knocked-down **(**Fig. 6F). Thus, while increased *p21*^*Cip1/Waf1*^ transcription may account for some of the increase in p21^*Cip1/Waf1*^ protein levels, it is unlikely the same is true for p27^*Kip1*^ protein levels. Taken together, these results show that the G_0_/G_1_ cell cycle defect is associated with the dysregulated expression of known G_1_ regulators, most notably increased protein levels of CDK inhibitors.

## Discussion

The dysregulation of multiple transcription factors are critical for the pathobiology of cHL, and in this study we show that the related AP-1 family transcription factors, c-Jun and JunB, each have important roles in promoting proliferation in this lymphoma. Knocking down either c-Jun or JunB expression was sufficient to reduce the growth rate of cHL cell lines *in vitro* and *in vivo*, which we demonstrate was a consequence of an impaired ability to transit through G_1_. Furthermore, we show that JunB knock-down also resulted in a similar cell cycle defect in the majority of ALK+ ALCL cell lines examined, whereas significant c-Jun knock-down in these cells had no effect on proliferation.

The largely similar G_0_/G_1_ cell cycle defect associated with c-Jun or JunB knock-down in cHL cell lines is intriguing (Fig. 3 and Table 1), and suggests that these transcription factors may regulate proliferation in HRS cells by a common mechanism(s). In keeping with this notion, the CDK inhibitor, p21^*Cip1/Waf1*^, was the only cell cycle regulator examined that was consistently altered in c-Jun or JunB knock-down cHL cell lines **(**Fig. 6A,B, results not shown), although the results were not as reproducibly convincing in c-Jun#2-1 shRNA–expressing cells (results not shown). While we believe that increased p21^*Cip1/Waf1*^ expression is a likely contributor to the proliferation defect, as up-regulation of this protein is linked to G_1_ arrest in multiple other cell types (refs^42–44^ as examples), we cannot rule out the involvement of other G_1_ regulators not investigated in this study.

We also observed a similar G_0_/G_1_ cell cycle defect in the majority of JunB knock-down ALK+ ALCL cell lines examined (Fig. 4); however, the molecular alterations differed from those observed in cHL cell lines. Inconsistent changes in p21^*Cip1/Waf1*^ expression were observed when JunB was knocked-down in Karpas 299 and SUP-M2 cells (Z. Wu and R.J. Ingham; unpublished results), but we observed a consistent up-regulation of p27^*Kip1*^ and p18^*Ink4C*^ protein levels, and a down-regulation of CDK2 levels in JunB shRNA–expressing Karpas 299 cells **(**Fig. 6C,D). p27^*Kip1*^ and CDK2 were similarly altered in SUP-M2 cells when JunB was knocked-down, and we also observed decreased Cyclin E expression in SUP-M2 cells **(**Fig. 6C,D). Importantly, increased p27^*Kip1*^ protein levels do not appear to be due to increased transcription **(**Fig. 6F), suggesting that the increased protein levels are a result of post-transcriptional regulation. Consistent with this idea, p27^*Kip1*^ is known to be regulated by post-transcriptional and post-translational mechanisms (reviewed in^45^). Likewise, p21^*Cip1/Waf1*^ is known to be regulated post-transcriptionally by miR-17/106b in cHL^46^, and can also be post-translationally regulated by multiple E3 ubiquitin ligases (reviewed in^47^). Thus, the increased p21^*Cip1/Waf1*^ protein levels observed in c-Jun/JunB knock-down cHL cell lines **(**Fig. 6A,B) may also be due to more than just increased *p21*^*Cip1/Waf1*^ transcription. Taken together, these results show that while a G_0_/G_1_ cell cycle defect was associated with reduced JunB expression in both cHL and ALK+ ALCL cell lines, the molecular basis for this defect may differ between these lymphomas.

An unaddressed question in this study is what dysregulated genes in c-Jun and/or JunB knock-down cells cause the G_0_/G_1_ cell cycle delay. We postulate that c-Jun/JunB may not be directly acting on the G_1_ regulators, but rather the observed phenotypes are likely due to the disruption of multiple genes/signalling pathways regulated by these transcription factors that converge on regulators of G_1_. One possibility is *CD30*. siRNA–mediated knock-down of CD30 in the SU-DHL-1 ALK+ ALCL cell line resulted in a decreased percentage of cells in S phase and increased percentages in G_1_^41^. In addition, treatment of cHL cell lines with CD30 siRNAs resulted in reduced viability^48^, but whether this was due to dysregulation of the cell cycle was not examined in this study. *CD30* is described as a JunB–specific target^19,30^ which might explain why reducing JunB, but not c-Jun, expression in ALK+ ALCL cell lines impaired proliferation (Figs 4 and 5). However, this also argues that the impaired proliferation observed in cHL cell lines expressing c-Jun shRNAs is not due to reduced CD30 expression (Figs 2 and 3). Thus, further work is needed to determine which dysregulated genes are responsible for the observed G_0_/G_1_ defects in the individual c-Jun/JunB knock-down cell lines. Moreover, we need to understand how the altered expression of these genes affects G_1_ regulators and which regulators, whether those identified in this study (Fig. 6**)** or others, account for the proliferation defect.

It is important to note that JunB knock-down did not reduce proliferation in the UCONN-L2 ALK+ ALCL cell line, and the proliferation defect associated with JunB knock-down was more severe in SUP-M2 than Karpas 299 cells (Fig. 4). Indeed, in SUP-M2 cells a significant portion of the JunB knock-down cells may be leaving the cell cycle (Fig. 4K). As mentioned, the effect of JunB knock-down also differed in cHL cell lines with the proliferation defect being more pronounced in L-540 and L-428 cells than KM-H2 cells (Figs 2 and 3; Table 1) further demonstrating that the importance of JunB in regulating proliferation varies amongst cHL and ALK+ ALCL cell lines. The variation in phenotype severity does not appear to be related to differences in JunB expression (Fig. 1 and Supplementary Fig. S6), nor the degree of JunB silencing (Figs 1 and 4) between cell lines. It is possible that these distinctions are due to different roles for JunB and/or the ability of other AP-1 proteins to compensate for reduced JunB expression in the individual cell lines.

In contrast to JunB, c-Jun knock-down had no effect on the growth rate of any of the ALK+ ALCL cell lines tested (Fig. 5). Our findings are consistent with siRNA knock-down experiments reported by Staber *et al*.^22^, but not with studies from another group showing that siRNA–mediated knock-down of c-Jun impacts the growth of ALK+ ALCL cell lines^23,41^. It is unclear why different groups have observed different phenotypes, but our results clearly show that the growth rates of three ALK+ ALCL cell lines with significantly reduced c-Jun expression were not impaired.

Why c-Jun knock-down has such different effects on proliferation in cHL and ALK+ ALCL is an important question arising from this study. Staber *et al*. reported that JunB mRNA is much more abundant than c-Jun mRNA in ALK+ ALCL cell lines^22^. If c-Jun and JunB functions are largely overlapping in ALK+ ALCL, differences in expression level could be a potential explanation for why JunB knock-down affects proliferation but c-Jun knock-down does not. However, since c-Jun and JunB protein levels are largely similar in cHL and ALK+ ALCL cell lines (Supplementary Fig. S6), this argues that there is also likely more JunB than c-Jun in cHL as well. Despite this, knock-down of c-Jun in cHL cell lines still impairs proliferation. Thus, the different outcomes associated with c-Jun knock-down in these two lymphomas could be a result of c-Jun regulating critical proliferation–associated genes in cHL that it does not in ALK+ ALCL, or that the amount of c-Jun present in the c-Jun knock-down cells is sufficient to promote proliferation in ALK+ ALCL but not cHL.

In summary, this study provides the first direct evidence that increased expression and/or activation of both c-Jun and JunB are involved in HRS cell proliferation by enabling cells to efficiently progress through G_1_ of the cell cycle. Moreover, the cell cycle defect was largely similar in cHL cell lines whether c-Jun or JunB was knocked-down. Finally, we show that stable JunB knock-down in the majority ALK+ ALCL cell lines tested resulted in a similar G_0_/G_1_ proliferation defect, whereas c-Jun knock-down had no effect on proliferation in any of the ALK+ ALCL cell lines examined. This indicates that while the role of JunB in promoting proliferation is largely similar in cHL and ALK+ ALCL cell lines, the importance of c-Jun in regulating proliferation differs between these two CD30–positive lymphomas.

## Methods

### Cell lines

The Epstein-Barr virus–negative cHL cell lines (KM-H2, L-428, and L-540) and ALK+ ALCL cell lines (Karpas 299 and SUP-M2) were obtained from the Leibniz Institute DSMZ-German Collection of Microorganisms and Cell Cultures (Braunschweig, Germany). The L-428 and L-540 cells are of nodular sclerosis cHL subtype, whereas KM-H2 cells are of the mixed cellularity subtype^37^. L-428 cells used in the mouse xenograft studies were obtained from Dr. Hesham Amin (The University of Texas, MD Anderson Cancer Center, Houston, TX). These cells were confirmed to be L-428 cells by short tandem repeat analysis and the effect on c-Jun and JunB knock-down in these cells was comparable to cells obtained from the DSMZ. The UCONN-L2 ALK+ ALCL cell line was obtained from Dr. Raymond Lai (University of Alberta; Edmonton, AB, Canada). The L-428, Karpas 299, SUP-M2, and UCONN-L2 cells were grown in RPMI 1640 media supplemented with 10% FBS, 2 mM L-glutamine (Gibco; Burlington, ON, Canada), 1 mM sodium pyruvate (Sigma-Aldrich; St Louis, MO), and 50 µM 2-mercaptoethanol (BioShop; Burlington, ON, Canada). KM-H2 and L-540 cells were grown in similar media with the exception that FBS was used at 20%. HEK 293T cells (American Type Culture Collection; Manassas, VA) were cultured in DMEM supplemented with 10% FBS, 1 mM sodium pyruvate and 2 mM L-glutamine. All cells were maintained at 37 °C in a 5% CO_2_ atmosphere.

### Antibodies

The anti-c-Jun antibody (60A8), anti-CDK2 (78B2), anti-p18^*INK4c*^ (DCS118), anti-p21^*Cip1/Waf1*^ (12D1), and anti-p27^*Kip1*^ (D69C12) antibodies (Abs) were purchased from Cell Signalling Technologies (Danvers, MA). The anti-JunB (C-11), anti-β-actin (AC-15), anti-α-tubulin (DM1A), and cyclin E (E4) Abs were from Santa Cruz Biotechnology (Santa Cruz, CA). The anti-GFP antibody (ab290) was from Abcam (Cambridge, MA), and the anti-Ki-67-PE (SolA15) antibody and FITC-conjugated goat anti-rabbit F(ab)’ were from Affymetrix eBioscience (Santa Clara, CA). Horseradish peroxidase-conjugated goat anti-rabbit IgG (H + L) and goat anti-mouse IgG (H + L) were from Jackson ImmunoReseach Laboratories (West Grove, PA). The anti-ALK (ALK1) Ab was from Dako (Glostrup, Denmark).

### Generation of shRNA–expressing cell lines

The MISSION^**®**^ shRNA lentiviral system (Sigma-Aldrich; St. Louis, MO) was used to generate lentiviral particles as previously described^49^. The shRNA constructs used are as following: non-targeting control shRNA SHC002 (control 002) (G418-resistant version for the xenograft studies and puromycin resistant version in all other experiments), non-targeting control shRNA SHC216 (control 216), c-Jun shRNA#1 (TRCN0000010366; G418-resistant version for the xenograft studies and puromycin resistant version in all other experiments), c-Jun shRNA#4 (TRCN0000039590), c-Jun shRNA#5 (TRCN0000039591), c-Jun shRNA#2-1 (TRCN0000355645), c-Jun shRNA#2-3 (TRCN0000355647), JunB shRNA#1 (TRCN0000014943; G418-resistant version for the xenograft studies and puromycin resistant version in all other experiments), JunB shRNA#3 (TRCN0000014945), JunB shRNA#5 (TRCN0000014947) and JunB shRNA#6 (TRCN0000232087). Of note, SHC002 is predicted not to target any known mammalian genes, whereas SHC216 is predicted not to target any known gene from any species^50^. Cells were placed in selection media containing 0.5 μg/ml puromycin or 750 μg/ml G418 (cells used in xenograft studies only) and experiments were performed between 1–3 weeks after selection.

### Western blotting experiments

Cells were lysed in 1% NP-40 lysis buffer^51^, detergent-insoluble material was removed by centrifugation, and protein concentrations were determined using the bicinchoninic acid protein assay kit (Thermo Scientific). Samples were run on SDS-PAGE gels, transferred to nitrocellulose membranes (Bio-Rad; Hercules, CA), and the membranes were then probed with the indicated primary antibodies. Blots were then incubated with horseradish-peroxidase–conjugated secondary antibodies and bands were visualized by exposing the membranes to enhanced chemiluminescence reagent (Thermo Scientific) and imaging either on film or an ImageQuant LAS 4000 imager (GE Healthcare Life Sciences; Mississauga, ON, Canada). The images in Fig. 6A were generated on a Li-Cor Odyssey Imager (Li-Cor Biotechnology; Lincoln NE) after probing blots with a fluorescent secondary Ab. Images were imported into PhotoShop (Adobe Systems Incorporated; Santa Clara, CA) where images were cropped. Images on blue film were also converted to grayscale. For reprobing, blots were stripped in TBS containing 0.1% Tween-20, pH 2 before being reprobed. All raw images are available in a Supplemental file.

### Growth Curve Experiments

Cells were resuspended to 4 × 10^4^ cells/ml (KM-H2), 5 × 10^4^ cells/ml (Karpas 299, SUP-M2, UCONN-L2), or 8 × 10^4^ cells/ml (L-428, L-540) in fresh media, and the number of viable cells was quantified daily by trypan blue exclusion using a hemocytometer. Doubling times were determined using methods adapted from Korzynska *et al*.^52^.

### BrdU and 7-AAD staining

BrdU (Bromodeoxyuridine) and 7-AAD (7-aminactinomycin D) staining was performed using the BD Pharmingen FITC or APC-BrdU Flow kit (BD Biosciences, San Jose, CA). Briefly, cells were incubated in the presence of 10 µM BrdU for 30 min (Karpas 299) or 1 h (KM-H2, L-428, SUP-M2, L-540) and processed as described in the manufacturer’s protocol. Data was collected on a BD LSR Fortessa flow cytometer (BD Biosciences) and analyzed using FlowJo software (Ashland, OR).

### Mouse xenograft experiments

The mouse studies were approved by the University of Alberta Animal Care and Use Committee (AUP00000393) and all experiments were performed in accordance with Canadian Council on Animal Care guidelines. 5 × 10^5^ L-428 cells expressing control, c-Jun, or JunB shRNA were resuspended in 50 μl of PBS and mixed with an equal volume of BD Matrigel^**®**^ Matrix (BD Bioscience). The cell/matrigel mixture was then injected subcutaneously into the neck of anesthetized two month old female NOD.CB17-*Prkdc*^*scid*^/J mice (Charles River; Montreal, QC, Canada). When palpable tumours of >1 cm in any direction were identified in any of the mice, the cohort was euthanized and tumours were harvested. Excised tumours were weighed and pictures of median sized tumours were taken.

### Introduction of a JunB cDNA into Karpas 299 cells

5 × 10^6^ Karpas 299 cells were transfected with 5 µg of plasmid DNA (pcDNA3-EGFP-P2A or pcDNA3-EGFP-P2A-FLAG-JunB) using an Amaxa nucleofector (Lonza AG; Basel Switzerland; Program A30, Kit V). The latter plasmid expresses a fusion protein between EGFP and JunB linked by the self-cleaving P2A peptide^53^. Seventy-two hours post transfection, cells were labelled with BrdU for 1 h, followed by LIVE/DEAD^TM^ Fixable Dead Cell Stain (Invitrogen; Carlsbad, CA) for 30 min on ice in the dark. Cells were then stained with an anti-GFP antibody followed by a FITC-conjugated anti-rabbit F(ab)’ fragment to enable us to specifically examine BrdU incorporation in transfected cells. Flow cytometry data was collected and analyzed as described above.

### Ki-67 staining

Cells were labelled with BrdU and processed as described in the BrdU/7-AAD labeling section. After DNase treatment, cells were stained with an anti-Ki-67-PE conjugated antibody for 30 min at room temperature. Flow cytometry data was collected and analyzed as described above.

### Quantitative Reverse Transcriptase Polymerase Chain Reaction (qRT-PCR)

The extraction of RNA and reverse transcription were performed as previously described^54^, and quantitative PCR was performed using the PerfeCTa SYBR Green FastMix kit (Quanta Biosciences; Gaithersburg, MD) and analyzed on a CFX96 Real-Time PCR Detection System (Bio-Rad). Samples were analyzed in triplicate, normalized to β-actin, and relative expression levels were determined using the 2^−ΔΔ*C*^_T_ method^55^. Data were normalized to control shRNA-expressing cells which were set at 100%. Primers used included: p21^*Cip1/Waf1*^, forward: GGGACAGCAGGAGACCAT, reverse: CGGCGTTTGGAGTGGTAG; p27^*Kip1*^, forward: GACAAACAGCGGAAAATCTAC, reverse: CTGGGCTGCCTTGAGTC; β-actin, forward: AGAAAATCTGGCACCACACC, reverse: TAGCACAGCCTGGATAGCAA.

### Statistical analyses

*t* tests were performed in Excel (Microsoft, Redmond, WA) whereas analysis of variance (ANOVA) with Tukey’s *post-hoc* tests were performed using R, version 3.5.0 (the R foundation). The specific test used in each case is indicated in the figure legends. A *P* < 0.05 was considered statistically significant.

### Ethics approval and consent to participate

The mouse experiments were approved by the University of Alberta Animal Care and Use Committee (AUP00000393).

## Electronic supplementary material

Supplementary Information

## Data Availability

The datasets generated during and/or analysed during the current study are available from the corresponding author on reasonable request. Data sharing is not applicable to this article as no datasets were generated or analysed during the current study.
