# Supplementary material for: The c-Jun and JunB transcription factors facilitate the transit of classical Hodgkin lymphoma tumour cells through G1
Source: Sci Rep. 2018 Oct 30;8:16019. doi: 10.1038/s41598-018-34199-9 (PMC6207696; doi:10.1038/s41598-018-34199-9)
Supplement: Supplementary file 1 — Supplementary Information [file 41598_2018_34199_MOESM1_ESM.pdf]

The c-Jun and JunB transcription factors facilitate the transit of classical Hodgkin lymphoma tumour cells through G_1

Jingxi Zhang[1*], Zuoqiao Wu[1*], Anton Savin[1], Mihye Yang[1], Ying-Han R. Hsu[2], Eugeniu Jantuan[2], Julinor T.C. Bacani[2], and Robert J. Ingham[1,§]

[1]Department of Medical Microbiology and Immunology and Li Ka Shing Institute of Virology, University of Alberta, Katz Group Centre for Pharmacy and Health Research, University of Alberta, Edmonton, AB, T6G 2E1, Canada.

[2]Department of Laboratory Medicine and Pathology, University of Alberta, Edmonton, AB, T6G 2B7, Canada.

* These authors contributed equally to this work

[§]Corresponding author

Email addresses: JZ: jingxi2@ualberta.ca

ZW: zuoqiao@ualberta.ca

AS: savin@ualberta.ca

MY: mihye3@ualberta.ca

YHRH: yinghan@ualberta.ca

EJ: jantuan@ualberta.ca

JTCB: bacani@ualberta.ca

RJI: ringham@ualberta.ca

Supplementary Figure Legends

Figure S1 – Western blots, growth curves and cell cycle analysis with a distinct control shRNA. Western blots **(A-C)**, growth curves **(D)**, and cell cycle analysis **(E)** of the indicated cHL cell lines including a control shRNA distinct from shown in **Figures 1-3.** The growth curves represent the average and standard deviation of two independent experiments and the cell cycle represents the average and standard deviation of four independent experiments. ANOVA with Tukey's *post hoc* test was performed in **E**. Molecular mass markers (in kDa) are indicated to the left of western blots. Note: some of the KM-H2 cell cycle data is included in Figure 3.

Figure S2 –Representative flow plots of control and c-Jun/JunB shRNA–expressing cHL cell lines. A. Gating strategy used to delineate stages of the cell cycle. Representative plots of L-540 **(B)**, L-428 **(C)**, and KM-H2 **(D)** cells used to generate the results in **Figures 3A-F**.

Figure S3 – Apoptosis measurement in c-Jun/JunB knock-down cells. Apoptosis was measured by TUNEL using the In Situ Cell Death Detection Kit (Roche Applied Science). Positive controls are cells pre-treated with 300 μg/ml DNase, whereas negative controls are permeabilized cells incubated in label solution without TdT enzyme.

Figure S4 – Analysis of ALK+ ALCL cell lines with a distinct control shRNA and representative flow blots from Figure 4. Representative western blots showing c-Jun/JunB expression in Karpas 299 **(A)** and SUP-M2 **(B)** cells expressing two control

shRNAs (control 216 and GFP) and the indicated c-Jun/JunB shRNAs. Note: the GFP shRNA targets the GFP protein, which is not expressed in these cells, and is used as a control in these experiments. As well, the seed sequences of JunB#6 shRNA and JunB#1 shRNA overlap and are not distinct. Growth curves **(C)** and BrdU labeling **(D)** of Karpas 299 cells expressing two control shRNAs and the indicated c-Jun/JunB shRNAs. **E**, BrdU/7-AAD labelling of SUP-M2 cells expressing two control shRNAs and the indicated c-Jun/JunB shRNAs. The growth curves (**C**) represent the average and standard deviation of three independent experiments and the data in **D** and **E** represents the average and standard deviation of two independent experiments. ANOVA with Tukey's *post hoc* test was performed in **C**. ** $P<0.01$, *** $P<0.001$. Representative BrdU/7-AAD plots of Karpas 299 **(F)** or SUP-M2 **(G)** cells used to generate the results in **Figures 4G, 4H,** and **5G**.

Figure S5 – Introduction of a JunB cDNA into JunB shRNA–expressing Karpas 299 cells. A. Protocol for JunB reintroduction **B. i.** Flow plots illustrating the EGFP–positive population **ii.** Histogram plots showing BrdU staining within the EGFP–positive population **C.** Western blot of FLAG-JunB introduced into JunB shRNA–expressing cells.

Figure S6 – c-Jun and JunB levels in cHL and ALK+ ALCL cells used in this study. Western blots showing c-Jun, JunB, or ALK protein levels in lysates of cHL (L-540, L-428, KM-H2), ALK+ ALCL (Karpas 299, SUP-M2, UCONN-L2) and two non-CD30–positive lymphoma cell lines (Ramos and Jurkat).

Figure S1

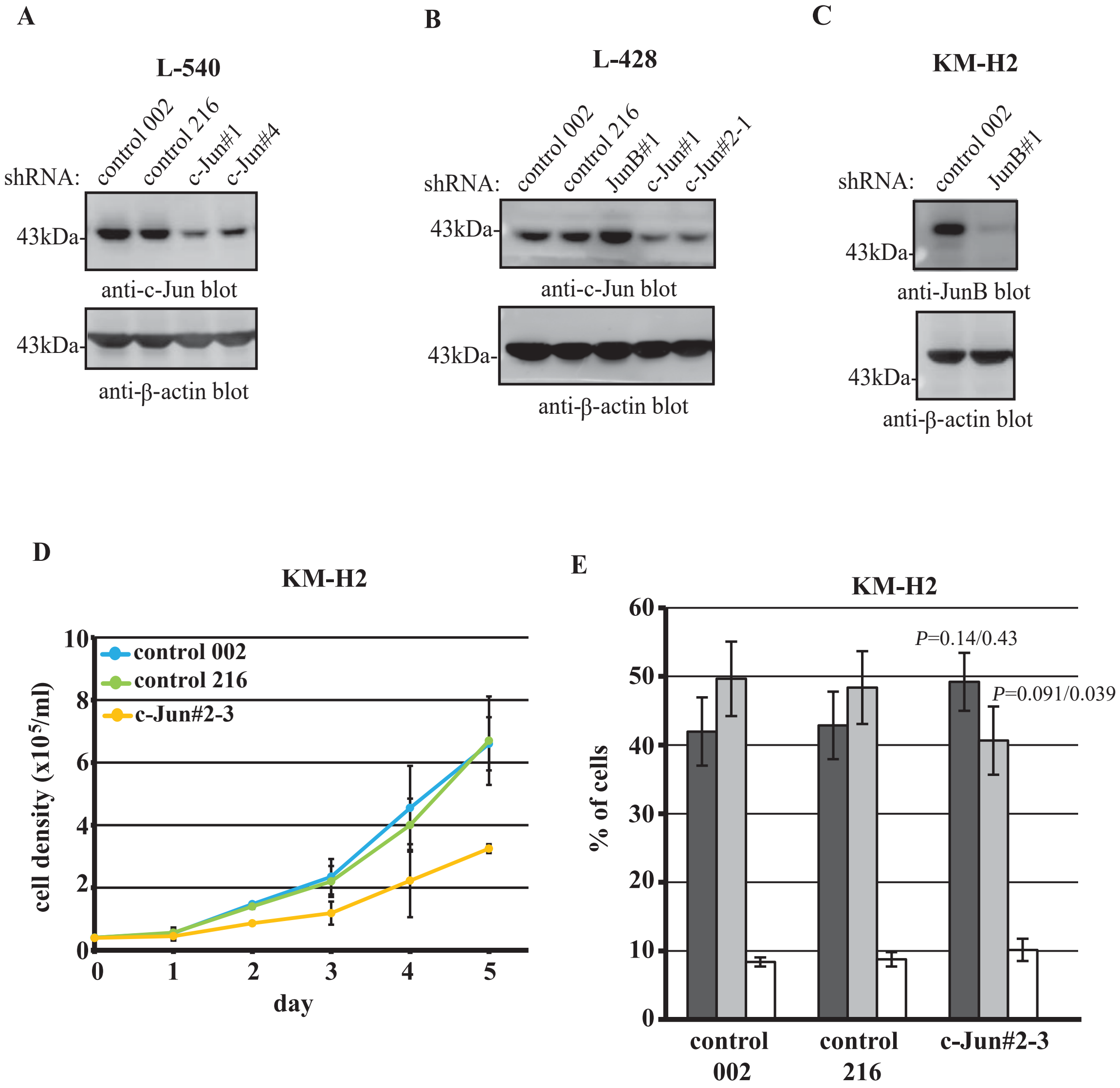

A
L-540
shRNA:
control 002
control 216
c-Jun#1
c-Jun#4
43kDa-
anti-c-Jun blot
43kDa-
anti-β-actin blot
B
L-428
shRNA:
control 002
control 216
JunB#1
c-Jun#1
c-Jun#2-1
43kDa-
anti-c-Jun blot
43kDa-
anti-β-actin blot
C
KM-H2
shRNA:
control 002
JunB#1
43kDa-
anti-JunB blot
43kDa-
anti-β-actin blot
D
KM-H2
control 002
control 216
c-Jun#2-3
cell density (x10^5/ml)
0
2
4
6
8
10
0
1
2
3
4
5
day
E
KM-H2
% of cells
0
10
20
30
40
50
60
P=0.14/0.43
P=0.091/0.039
control 002
control 216
c-Jun#2-3

Figure S2

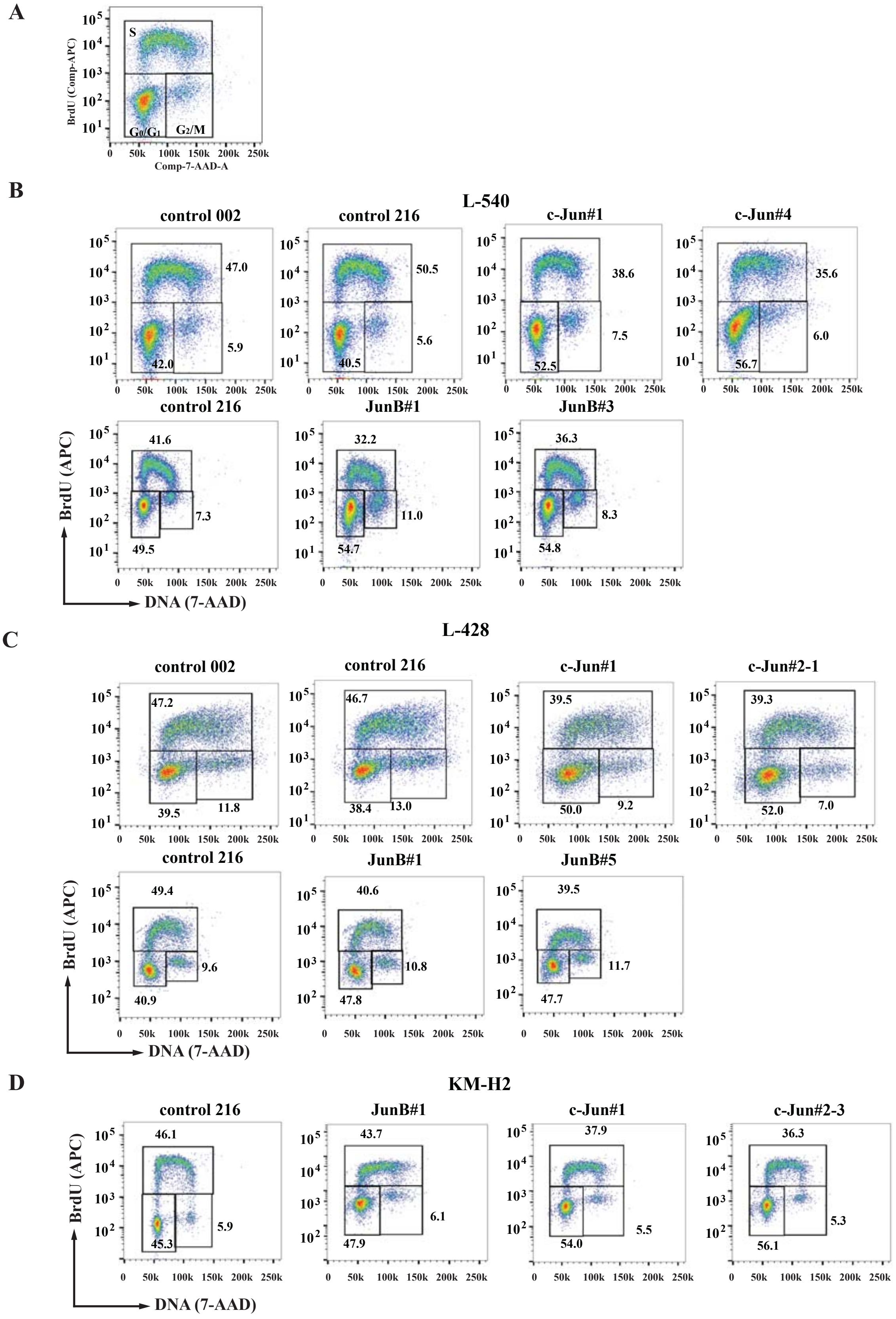
A
S
G0/G1
G2/M
BrdU (Comp-APC)
Comp-7-AAD-A
B
L-540
control 002
control 216
c-Jun#1
c-Jun#4
47.0
5.9
42.0
50.5
5.6
40.5
38.6
7.5
52.5
35.6
6.0
56.7
control 216
JunB#1
JunB#3
41.6
7.3
49.5
32.2
11.0
54.7
36.3
8.3
54.8
BrdU (APC)
DNA (7-AAD)
C
L-428
control 002
control 216
c-Jun#1
c-Jun#2-1
47.2
39.5
11.8
46.7
38.4
13.0
39.5
50.0
9.2
39.3
52.0
7.0
control 216
JunB#1
JunB#5
49.4
9.6
40.9
40.6
10.8
47.8
39.5
11.7
47.7
BrdU (APC)
DNA (7-AAD)
D
KM-H2
control 216
JunB#1
c-Jun#1
c-Jun#2-3
46.1
5.9
45.3
43.7
6.1
47.9
37.9
5.5
54.0
36.3
5.3
56.1
BrdU (APC)
DNA (7-AAD)

Figure S3

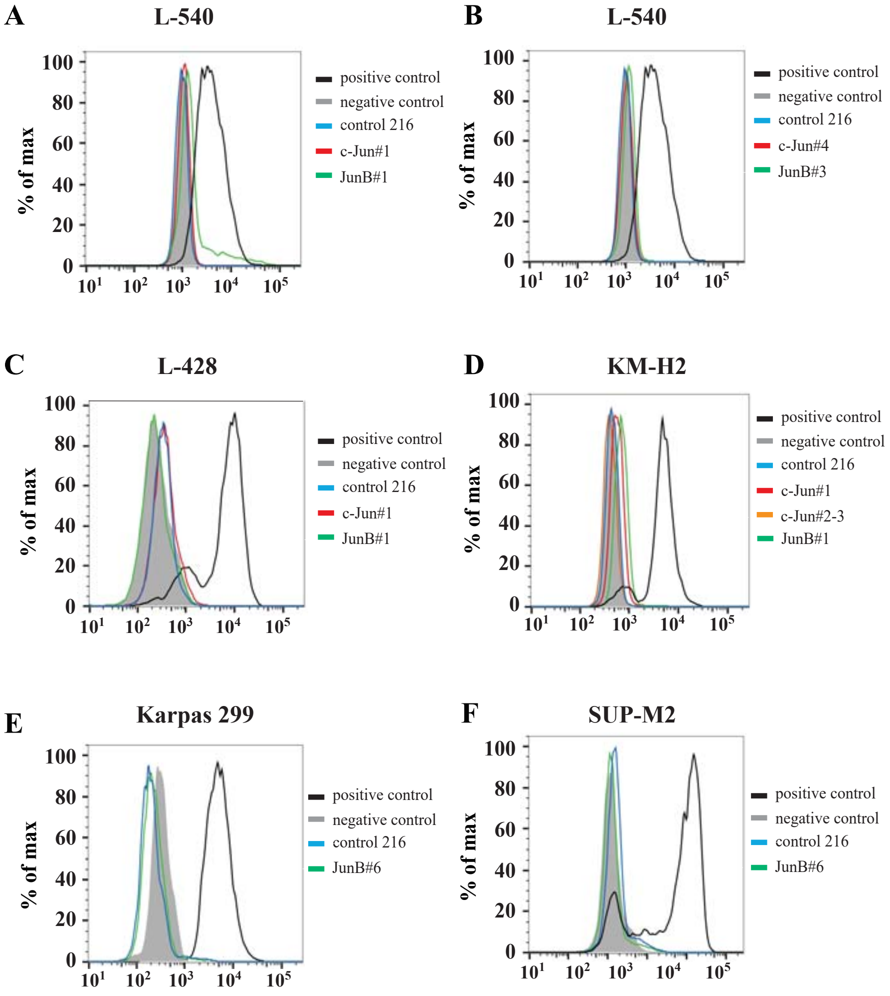

A
L-540
% of max
positive control
negative control
control 216
c-Jun#1
JunB#1
B
L-540
positive control
negative control
control 216
c-Jun#4
JunB#3
C
L-428
positive control
negative control
control 216
c-Jun#1
JunB#1
D
KM-H2
positive control
negative control
control 216
c-Jun#1
c-Jun#2-3
JunB#1
E
Karpas 299
positive control
negative control
control 216
JunB#6
F
SUP-M2
positive control
negative control
control 216
JunB#6

Figure S4

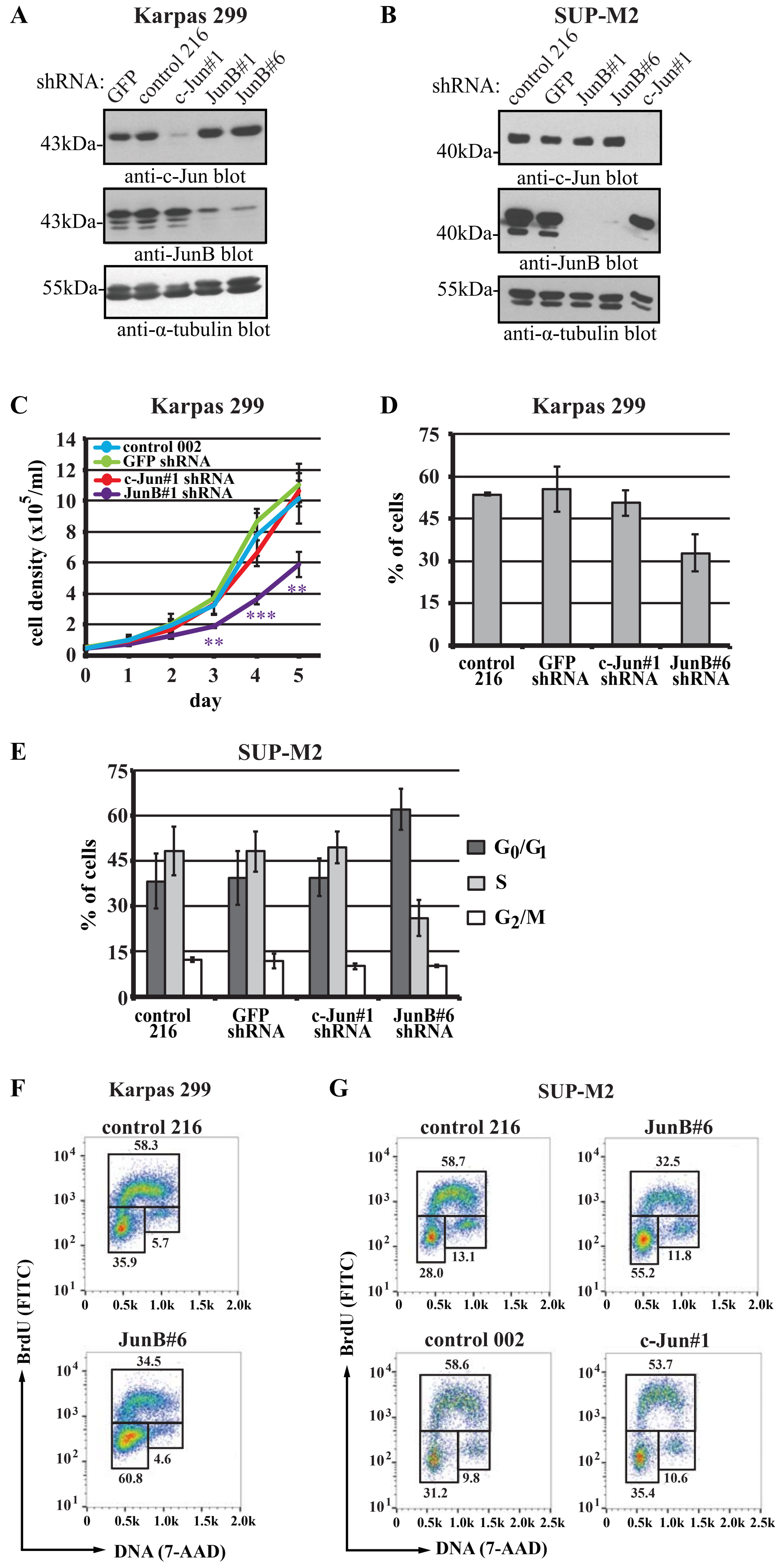
A
Karpas 299
shRNA: GFP control 216 c-Jun#1 JunB#1 JunB#6
43kDa-
anti-c-Jun blot
43kDa-
anti-JunB blot
55kDa-
anti-α-tubulin blot
B
SUP-M2
shRNA: control 216 GFP JunB#1 JunB#6 c-Jun#1
40kDa-
anti-c-Jun blot
40kDa-
anti-JunB blot
55kDa-
anti-α-tubulin blot
C
Karpas 299
control 002
GFP shRNA
c-Jun#1 shRNA
JunB#1 shRNA
cell density (x10^5/ml)
day
**
***
**
D
Karpas 299
% of cells
control 216
GFP shRNA
c-Jun#1 shRNA
JunB#6 shRNA
E
SUP-M2
% of cells
G0/G1
S
G2/M
control 216
GFP shRNA
c-Jun#1 shRNA
JunB#6 shRNA
F
Karpas 299
control 216
58.3
5.7
35.9
JunB#6
34.5
4.6
60.8
BrdU (FITC)
DNA (7-AAD)
G
SUP-M2
control 216
58.7
13.1
28.0
JunB#6
32.5
11.8
55.2
control 002
58.6
9.8
31.2
c-Jun#1
53.7
10.6
35.4
BrdU (FITC)
DNA (7-AAD)

Figure S5

A **Karpas 299**

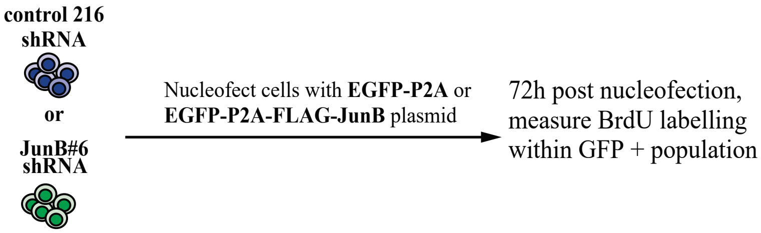

B

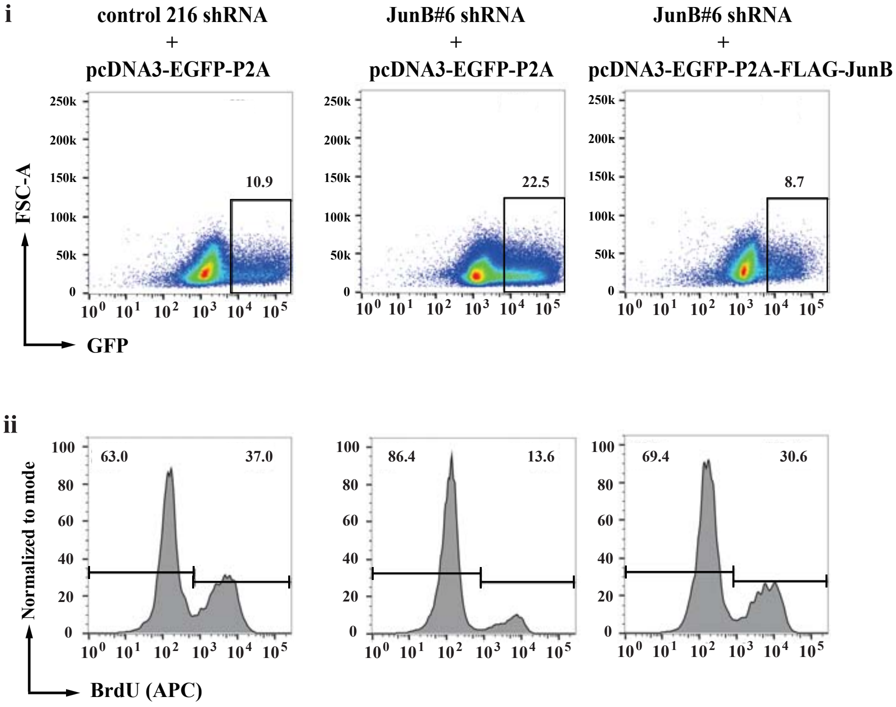

C **Karpas 299**

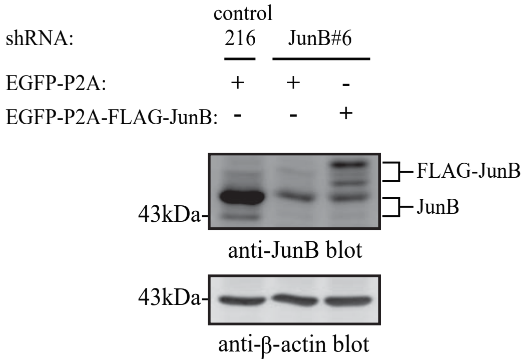

Figure S6

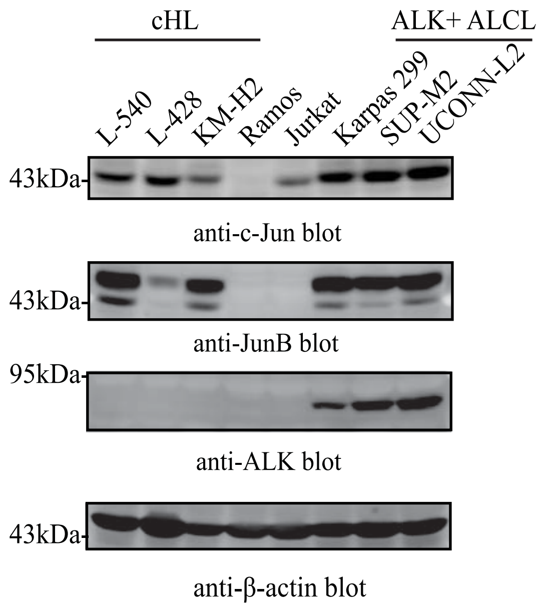
cHL
ALK+ ALCL
L-540
L-428
KM-H2
Ramos
Jurkat
Karpas 299
SUP-M2
UCONN-L2
43kDa
anti-c-Jun blot
43kDa
anti-JunB blot
95kDa
anti-ALK blot
43kDa
anti-β-actin blot

Raw Data for Figures 1A & B

A

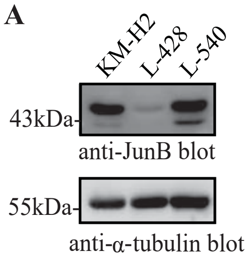

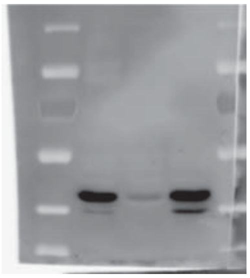

anti-JunB blot

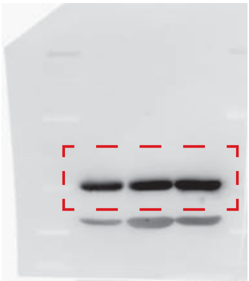

anti-α-tubulin blot

note: the lower band is a remnant of another antibody this blot was probed with prior to anti-α-tubulin

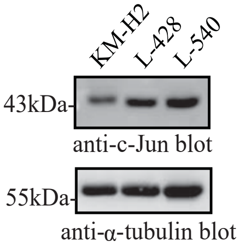

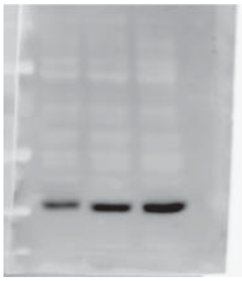

anti-c-Jun blot

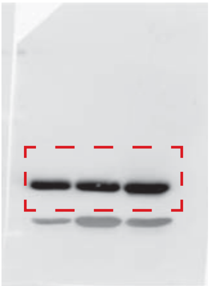

anti-α-tubulin blot

note: the lower band is a remnant of another antibody this blot was probed with prior to anti-α-tubulin

B

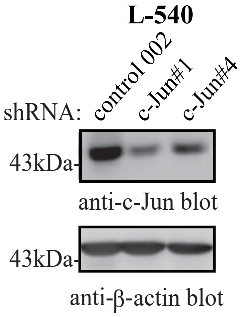

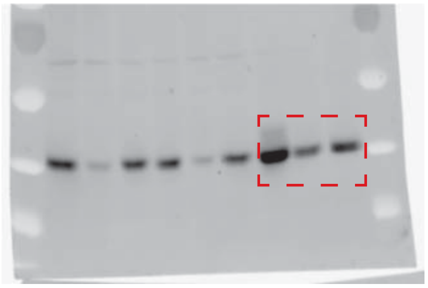

anti-c-Jun blot

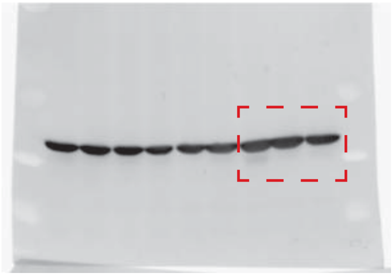

anti-β-actin blot

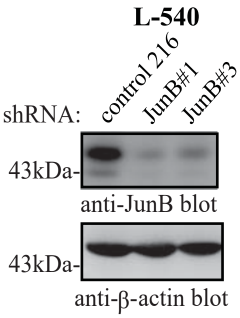

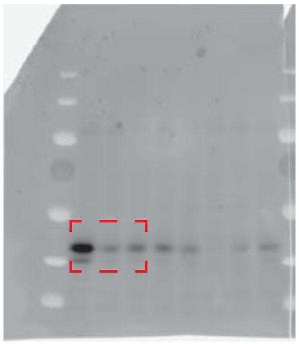

anti-JunB blot

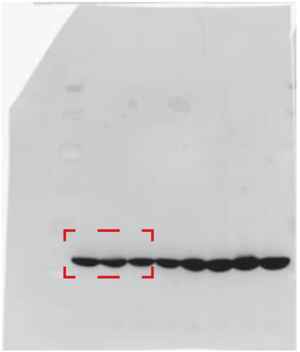

anti-β-actin blot

Raw Data for Figures 1C & D

C

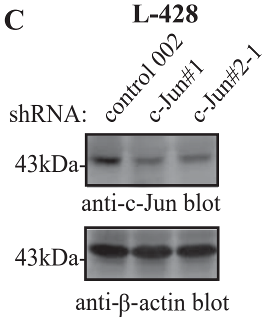

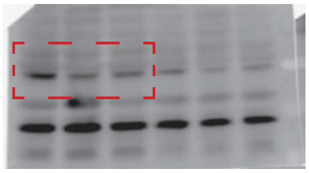

anti-c-Jun blot

note: the lower bands are remnants of other antibodies this blot was probed with prior to anti-c-Jun

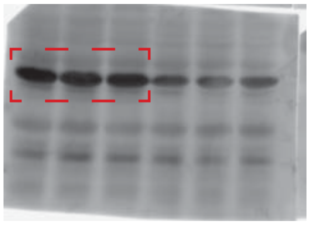

anti-β-actin blot

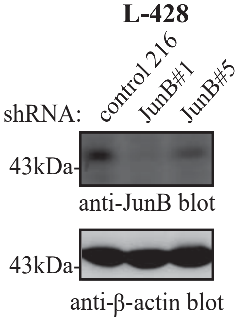

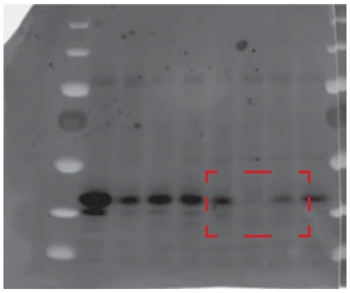

anti-JunB blot

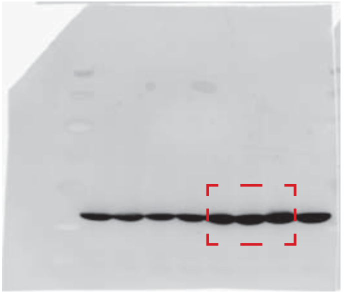

anti-β-actin blot

D

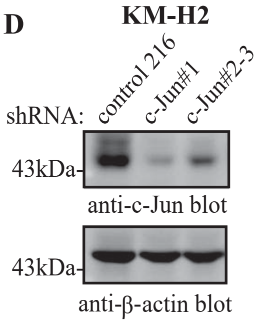

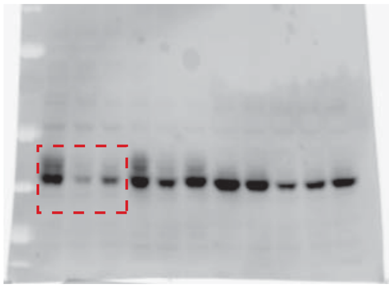

anti-c-Jun blot

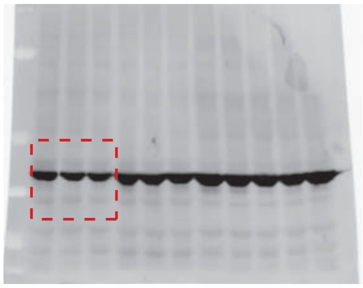

anti-β-actin blot

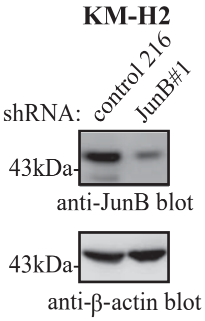

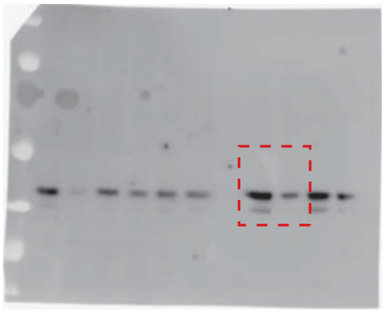

anti-JunB blot

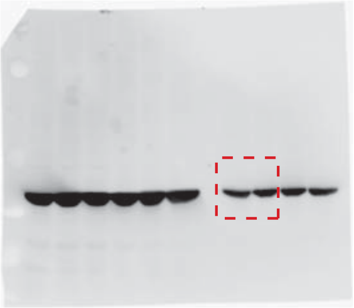

anti-β-actin blot

Raw Data for Figure 2

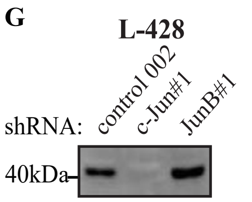

anti-c-Jun blot

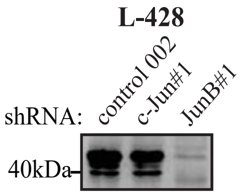

anti-JunB blot

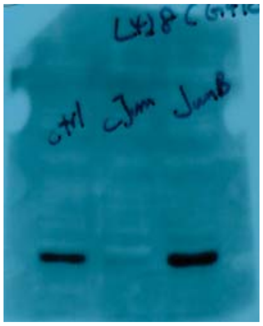

anti-c-Jun blot

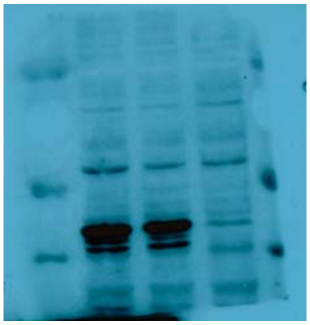

anti-JunB blot

Raw Data for Figure 4

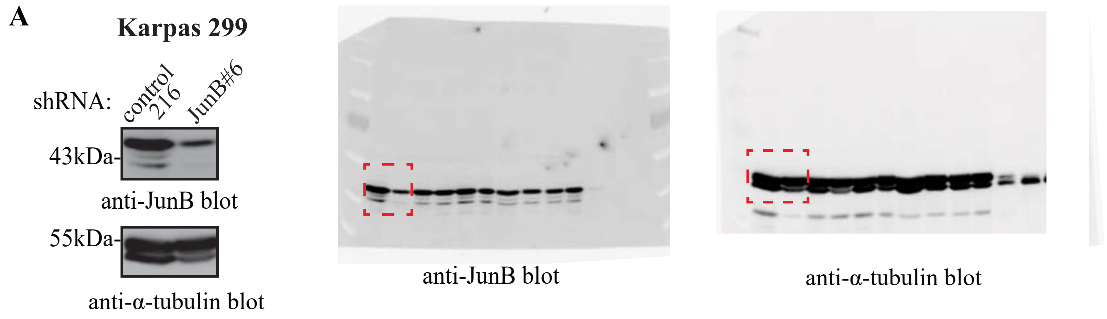

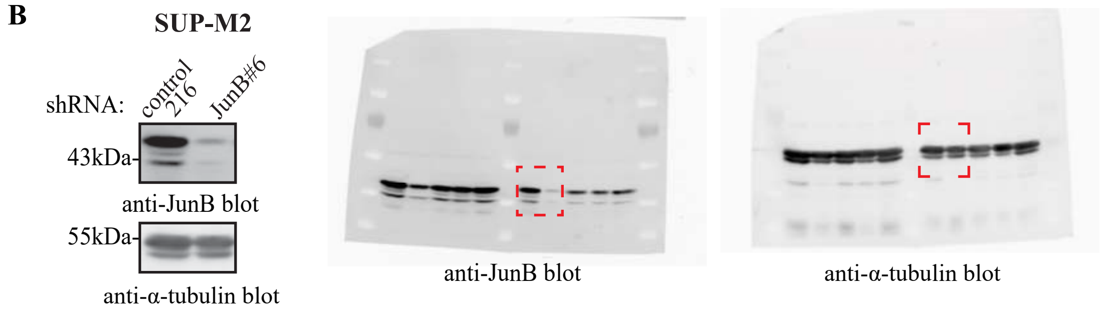

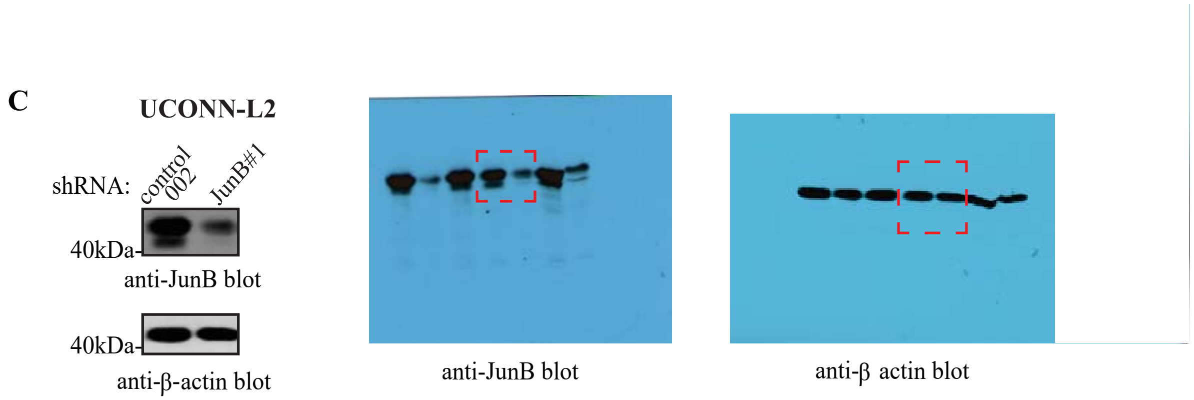

Raw Data for Figure 5

A **Karpas 299**

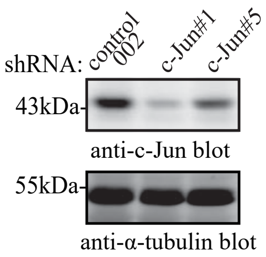

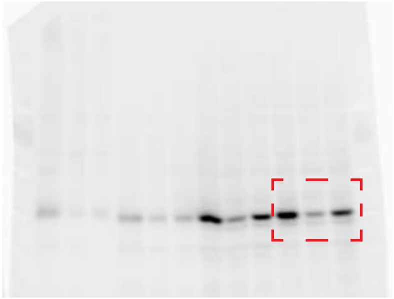

anti-c-Jun blot

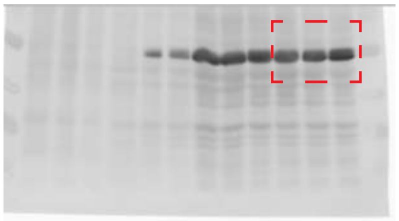

anti-α-tubulin blot

C **SUP-M2**

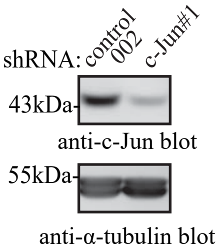

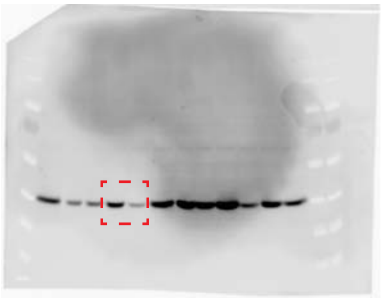

anti-c-Jun blot

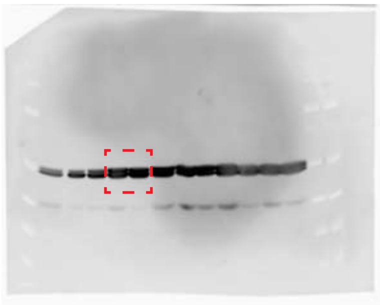

anti-α-tubulin blot

E **UCONN-L2**

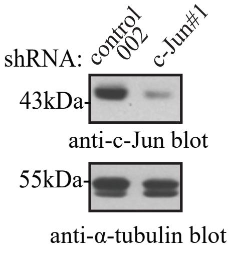

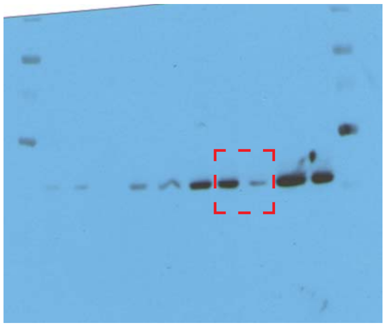

anti-c-Jun blot

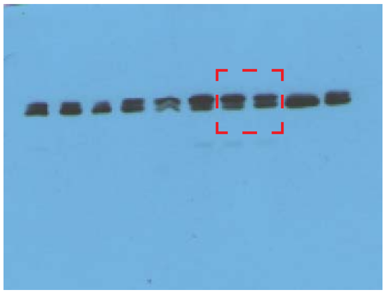

anti-α-tubulin blot

Raw Data for Figures 6A & B

A

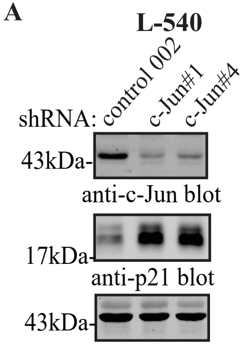

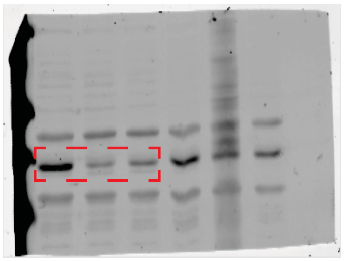

anti-c-Jun blot

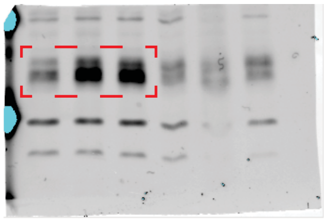

anti-p21 blot

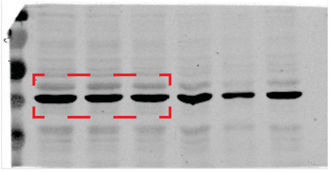

anti-β-actin blot

note: the additional bands are remnants of other antibodies this blot was probed with prior to anti-c-Jun

note: all blots are from the same membrane that was cut prior to probing

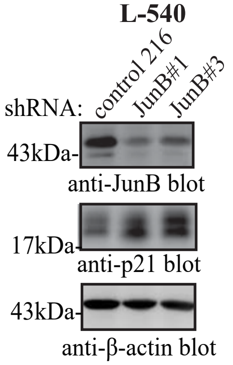

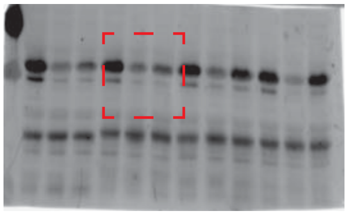

anti-JunB blot

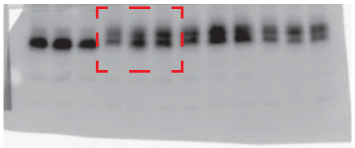

anti-p21 blot

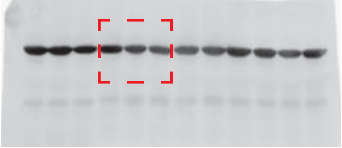

anti-β-actin blot

note: the lower band is a remnant of another antibody this blot was probed with prior to anti-c-Jun

note: all blots are from the same membrane that was cut prior to probing

B

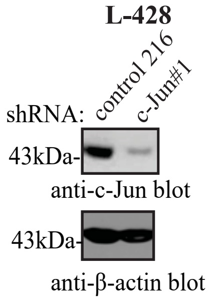

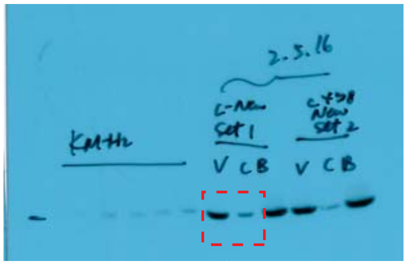

anti-c-Jun blot

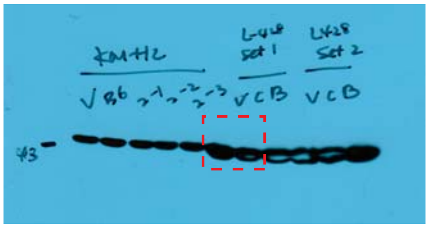

anti-β-actin blot

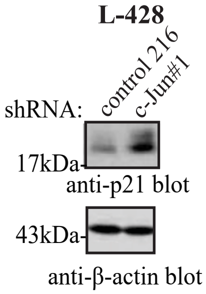

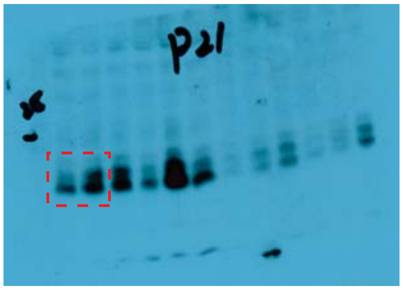

anti-p21 blot

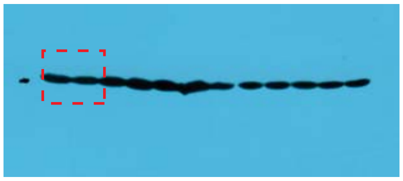

anti-β-actin blot

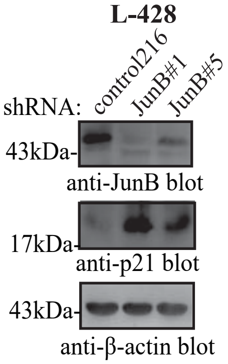

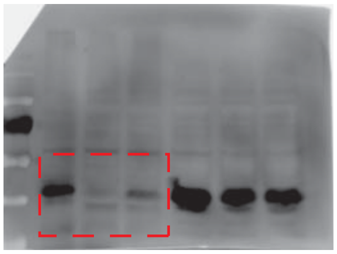

anti-JunB blot

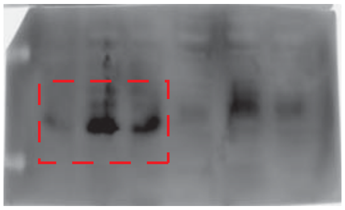

anti-p21 blot

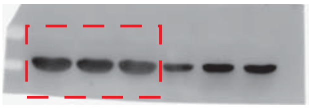

anti-β-actin blot

note: all blots are from the same membrane that was cut prior to probing

Raw Data for Figures 6C & D

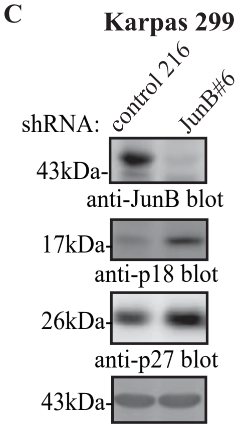

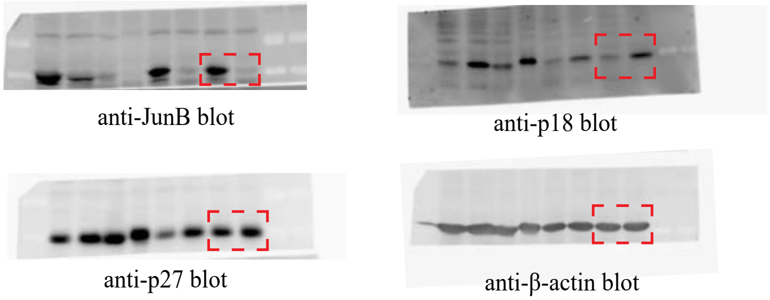

note: all blots are from the same membrane that was cut prior to probing

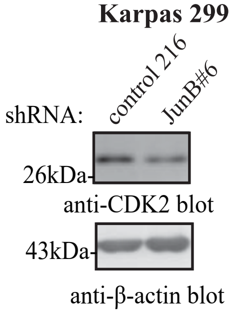

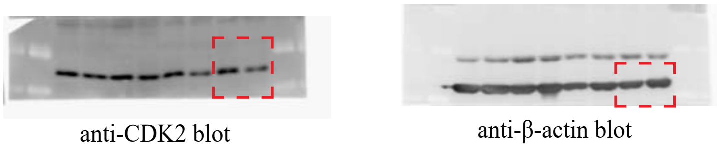

note: the upper blot band in anti-β-actin blot is a remnant from probing this membrane with another antibody

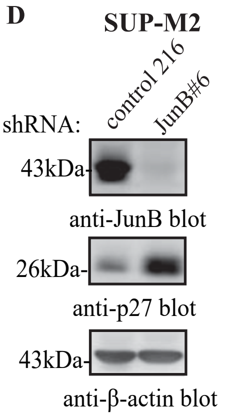

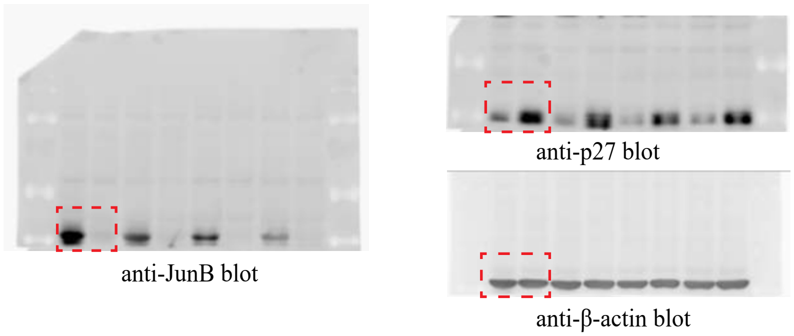

note: all blots are from the same membrane

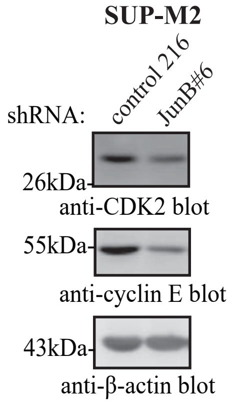

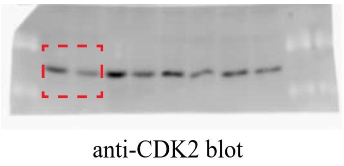

note: all blots are from the same membrane

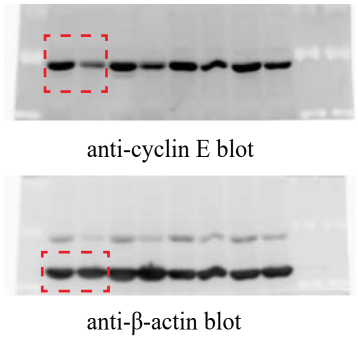

note: the upper blot band in anti-β-actin blot is a remnant from probing this membrane with another antibody

Figure S1 Raw Data

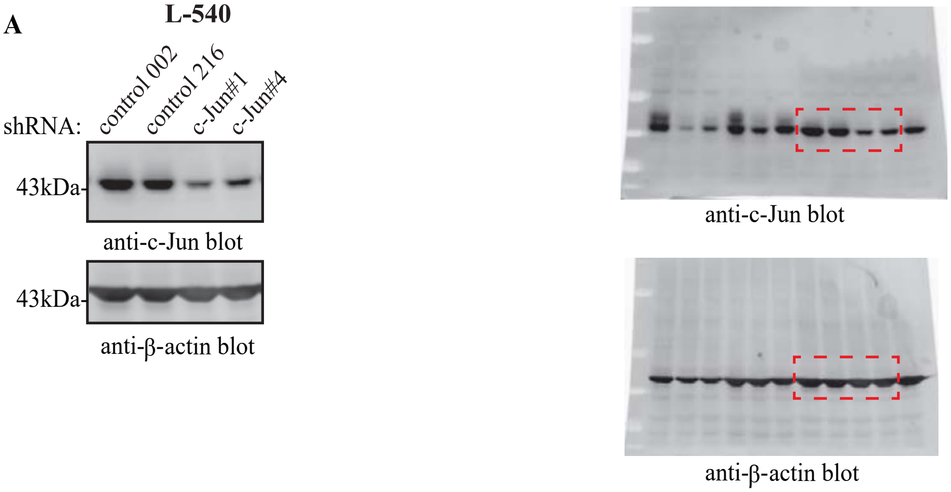

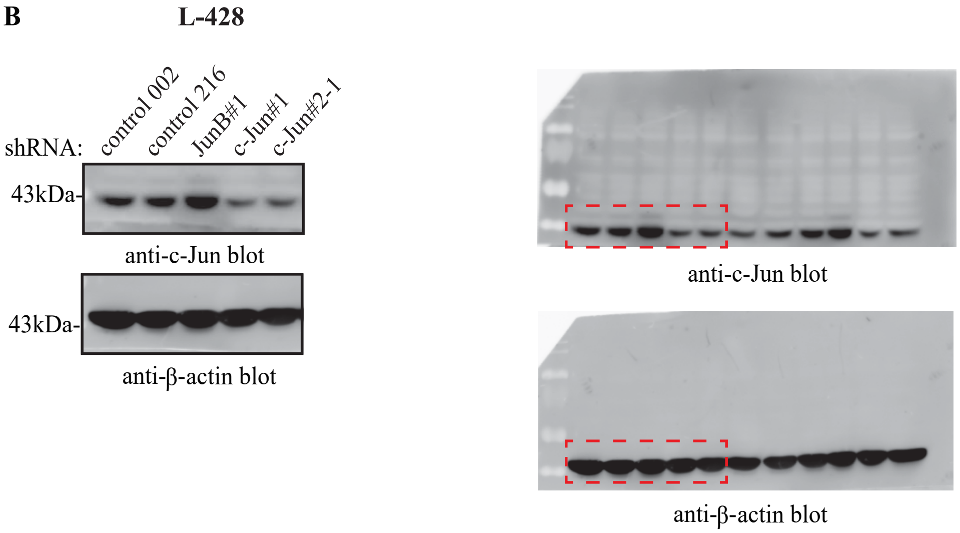

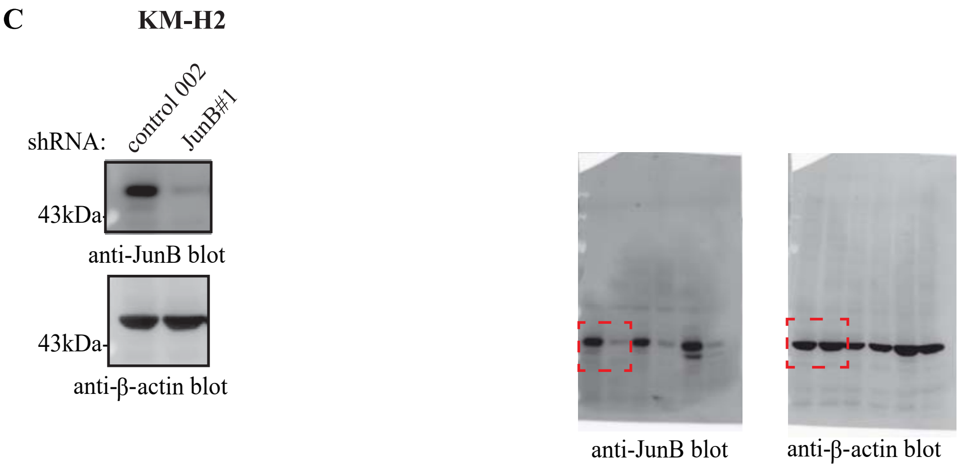

Figure S4 RawData

A **Karpas 299**

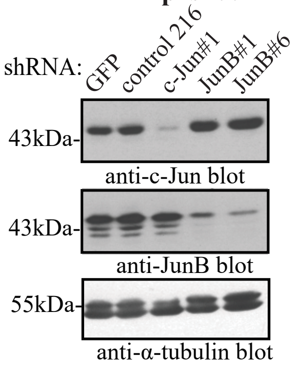

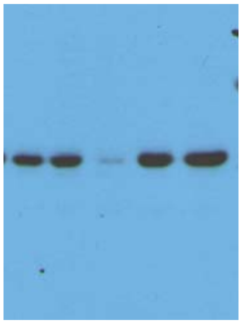

anti-c-Jun blot

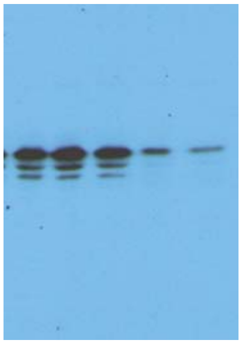

anti-JunB blot

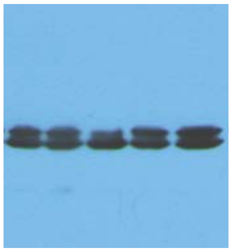

anti-α-tubulin blot

B **SUP-M2**

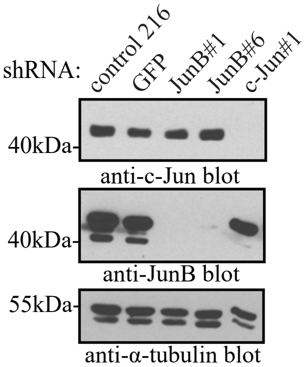

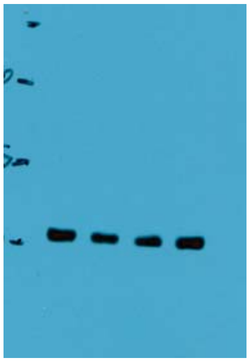

anti-c-Jun blot

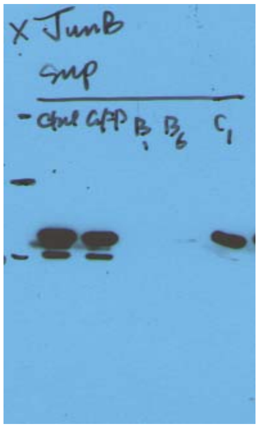

anti-JunB blot

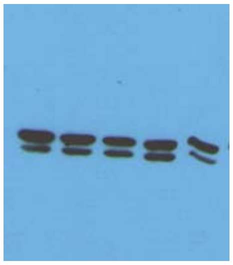

anti-α-tubulin blot

Raw Data for Figure S5

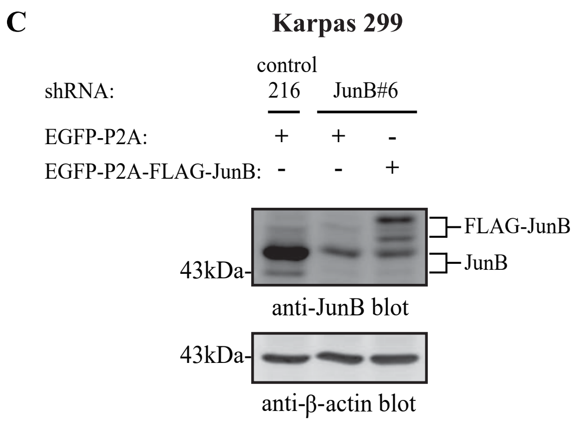

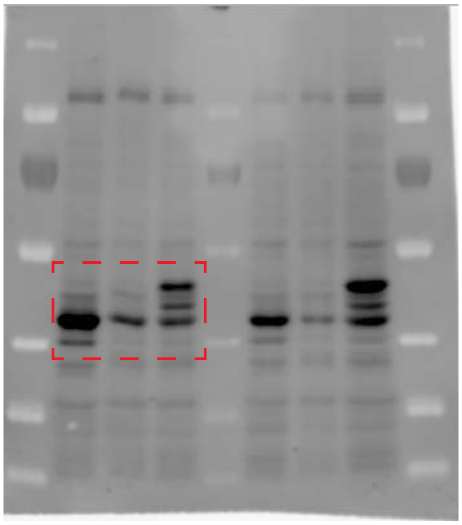

anti-JunB blot

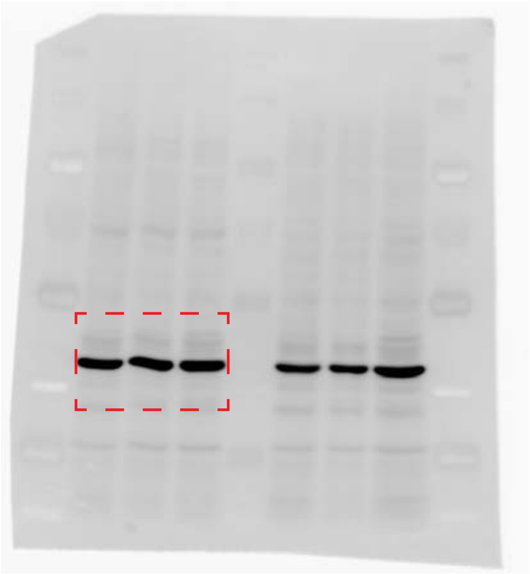

anti-β-actin reprobe

Raw Data for Figure S6

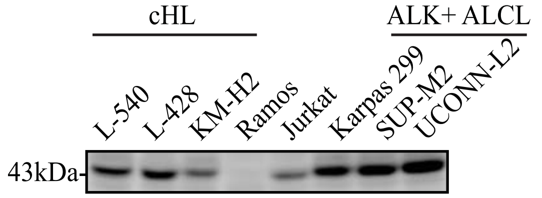

anti-c-Jun blot

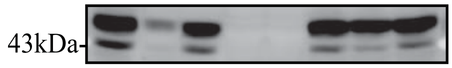

anti-JunB blot

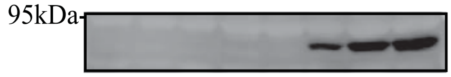

anti-ALK blot

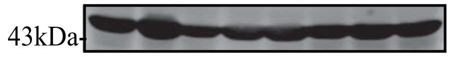

anti-β-actin blot

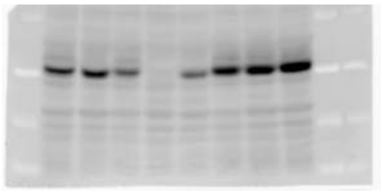

anti-c-Jun blot

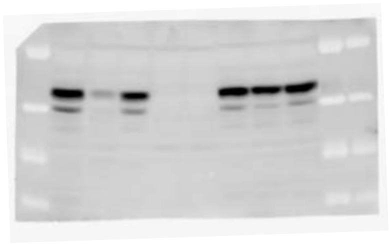

anti-JunB blot

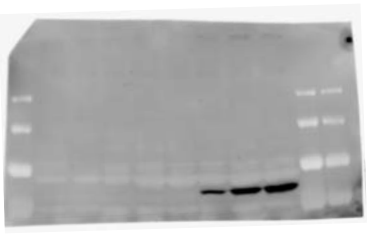

anti-ALK blot

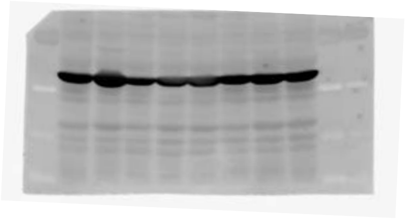

anti-β-actin blot

note: all blots are from the same membrane
